# Nutritional selenium status in a cohort of university students: impact on intellectual and cognitive performance

**DOI:** 10.3389/fnut.2026.1829412

**Published:** 2026-06-05

**Authors:** Martínez-García Rosa María, Dimas-Benedicto Carmen, Albasanz Jose Luis, Castro-Vázquez Lucía, Blázquez-Abellán Gema, Aparicio Aránzazu, Martín Mairena

**Affiliations:** 1 NUTRI-SAF Research Group, Department of Nursing, Physiotherapy and Occupational Therapy, Faculty of Nursing, University of Castilla-La Mancha, Cuenca, Spain; 2 Department of Inorganic, Organic and Biochemistry, Faculty of Medicine of Ciudad Real/Faculty of Chemical Sciences and Technologies, University of Castilla-La Mancha, Cuenca, Spain; 3 Institute of Biomedicine (IB-UCLM), University of Castilla-La Mancha, Albacete, Spain; 4 Grupo de Neuroquímica de CR, Instituto de Investigación Sanitaria de Castilla-La Mancha (IDISCAM), Toledo, Spain; 5 NUTRI-SAF Research Group, Department of Analytical Chemistry and Food Technology, Faculty of Pharmacy, University of Castilla-La Mancha, Albacete, Spain; 6 NUTRI-SAF Research Group, Department of Medical Science, Faculty of Pharmacy, University of Castilla-La Mancha, Albacete, Spain; 7 Department of Nutrition and Food Science, Complutense University of Madrid, Madrid, Spain; 8 VALORNUT Research Group, Department of Nutrition and Food Science, Faculty of Pharmacy, Complutense University of Madrid, Madrid, Spain; 9 San Carlos Health Research Institute (IdISSC), Madrid, Spain

**Keywords:** cognitive, dietary intake, energy, selenium, serum

## Abstract

**Introduction:**

Evidence on selenium status and cognitive performance in university populations remains limited.

**Objectives:**

We examined dietary intake and serum selenium concentration, and their associations with cognitive performance in Spanish university students.

**Methods:**

A cross-sectional study was conducted in 132 students aged 18–29 years. Participants completed a dietary record to estimate selenium intake. Cognitive performance was assessed using the WAIS-IV, including the Total Intelligence Quotient (mean TIQ = 90) and its cognitive indices. Serum selenium status was classified according to a reference adequacy threshold of 80 μg/L. Associations between selenium status and cognitive outcomes were evaluated using Spearman correlations and linear regression models.

**Results:**

The 53.5% of female and 16.4% of male fell below the reference serum value. Students with TIQ < 90 had lower serum selenium than those with TIQ ≥ 90 (84.9 ± 16.9 vs. 93.3 ± 21.4 μg/L). Serum selenium was positively correlated with TIQ, Verbal Comprehension index “VCI” and Working Memory “WMI.” Dietary selenium intake correlated with VCI. In adjusted models, male sex and higher energy intake were positively associated with TIQ, whereas selenium intake showed an inverse association with TIQ (*β* = −0.067 points/μg/day) and Perceptual Reasoning Index “PRI” (*β* = −0.129 points/μg/day), and was not independently associated with VCI, WMI, or Processing Speed Index “PSI.”

**Conclusion:**

Selenium intake and serum selenium were associated with cognitive outcomes in unadjusted analyses, while adjusted models suggested a more complex relationship. A substantial proportion of female exhibited low serum selenium levels, underscoring the importance of monitoring selenium status in young adults and warranting confirmation in longitudinal studies.

## 1 Introduction

Selenium (Se) is an essential micronutrient required for the synthesis of selenoproteins involved in key processes such as antioxidant defense and redox regulation, with potentially relevant effects on Immune regulation (1) tissue homeostasis and neurological function (2, 3). In humans, 25 genes encoding selenoproteins have been identified (4).

Among the major antioxidant selenoproteins are glutathione peroxidases (GPX) and thioredoxin reductases (TXNRD), which help limit oxidative damage through peroxide reduction and the regeneration of cellular antioxidant systems (5, 6). Interest in Se in relation to cognitive function is partly supported by the vulnerability of the central nervous system to oxidative stress. The brain has a high oxygen demand and elevated metabolic activity, which promotes the generation of reactive species that, in excess, may impair neuronal integrity and synaptic plasticity, that remains highly active in young adulthood, with potential negative consequences for learning, memory, and cognitive performance (7, 8). In this context, Se has been proposed to exert neuroprotective actions through selenoproteins involved in the control of oxidative stress and neuroinflammation, among other pathways (3, 9). However, the exact role of Se in cognitive function remains to be fully elucidated, and proposed mechanisms include modulation of redox balance, regulated cell-death processes such as ferroptosis, and pathways related to neuronal signaling and homeostasis (3, 10). Although selenium participates in antioxidant defense and may exert neuroprotective effects (11), evidence also suggests that excessive selenium exposure or certain selenium species may have adverse neurological consequences (12). A U-shaped association has been described between selenium status and cognitive outcomes, with increased risk of cognitive decline or progression from mild cognitive impairment to dementia reported at both low and high selenium levels (13). These findings highlight the complexity of selenium biology and the importance of considering dose, chemical form, and population characteristics when interpreting its effects on cognitive function (14).

Globally, selenium deficiency shows wide geographical variability due to differences in soil content, dietary patterns, and the cut-off criteria used to define deficiency. Estimates indicate that deficiency is more prevalent in regions with low soil selenium, such as parts of Europe, China, and sub-Saharan Africa. These variations highlight the importance of contextualizing selenium status within regional dietary and environmental conditions (15–17). This complexity is relevant for interpreting potential non-linear or unexpected associations observed in epidemiological studies.

In Spain, although population-based evidence in young people is limited, data in children suggest that apparently adequate dietary intake does not always translate into optimal serum Se status. In schoolchildren from the Madrid region (8–13 years), mean Se intake was ~91 μg/day and most participants met recommended intakes; however, ~14% showed serum Se concentrations <60 μg/L, and the correlation between intake and serum Se was weak (18). Additionally, in another study in Madrid schoolchildren, excess body weight was associated with lower serum Se concentrations and a higher probability of deficiency, highlighting the importance of considering anthropometric and lifestyle variables when studying Se status (19).

Several studies have reported associations between Se status and cognitive performance across different life stages. In children, both maternal Se status during pregnancy and early-life Se status have been associated with neuropsychological development and later cognitive outcomes in different cohorts (20–22). In older adults, lower Se levels have also been linked to poorer cognitive functioning and a higher risk of cognitive impairment (16, 23). Nevertheless, the available evidence remains heterogeneous depending on the biomarker used, the exposure range, confounding factors, and dietary context; therefore, further studies are needed in less explored populations and among individuals with stabilized cognitive capacity (9).

Accordingly, this study aimed to examine the association between dietary selenium intake and serum selenium concentration and intellectual and cognitive performance in young university students, including both total intelligence quotient (TIQ) and specific cognitive indices. To our knowledge, this is among the first studies to jointly evaluate selenium intake and serum status in relation to cognition in a university population.

## 2 Materials and methods

### 2.1 Study design and participants

This cross-sectional observational study included 132 university students (male and female) aged 18–29 years. Participants were recruited from the Faculties of Nursing, Business Administration and Management, Social Work, Fine Arts, Telecommunications Engineering, and Education at the University of Castilla-La Mancha (UCLM), Cuenca Campus (Spain). Data collection was conducted between June 2018 and December 2019. The study protocol was approved by the Ethics Committee of Virgen de la Luz Hospital (Cuenca, Spain) (REG: 2017/PI1417; approval date: 28 May 2018).

The sample size calculation was performed *a priori* based on the expected prevalence of selenium deficiency in young Spanish populations, as reported by López-Sobaler (24) which ranges between 12 and 15%. We used an expected prevalence of 15%, a 95% confidence level, a precision (margin of error) of 6%, and a statistical power of 80%. Using the standard formula for estimating a proportion in cross-sectional studies:


$$>n=\frac{Z^{2}\cdot p(1-p)}{d^{2}}>$$

(n): Sample size; (Z): Desired confidence level; (p): Prevalence; (d): Precision or margin of error.

The minimum theoretical sample size required was *n* = 136 participants. Our final sample included 132 students, which is very close to the calculated requirement and considered acceptable given the small number of exclusions.

Students were initially approached and verbally informed about the study and screened for eligibility. Inclusion criteria were: (i) age 18–29 years; (ii) current enrolment as a university student; (iii) provision of written informed consent; and (iv) absence of medical conditions that could substantially affect the interpretation of the results. Exclusion criteria were: (i) age >29 years; (ii) chronic diseases; and (iii) use of medications or substances that could interfere with cognitive assessment or study outcomes. Additionally, participants reporting alcohol consumption ≥50 g/week were excluded.

All eligible participants received a written information sheet describing the study rationale and procedures and signed an informed consent form. Participants could withdraw at any time without providing a reason.

The study was conducted in accordance with the Declaration of Helsinki and its later amendments. Personal data were handled confidentially and accessed only by authorized researchers. Data processing complied with the General Data Protection Regulation (EU) 2016/679 (25) and Spanish Organic Law 3/2018 on Personal Data Protection and Guarantee of Digital Rights (26).

### 2.2 Study procedures and variables

After signing informed consent, participants attended two sessions at the Faculty of Nursing (UCLM, Cuenca campus). During the first session, the following data were collected: sociodemographic and health-related information, physical activity, anthropometric measurements, and cognitive assessment. Dietary data were collected using a 4-day food and beverage record (including one Sunday or public holiday), which participants completed between visits and returned at the second session.

The second session took place within 20 days of the first visit and included collection of a fasting blood sample for hematological and biochemical analyses and retrieval/review of the dietary record.

### 2.3 Sociodemographic and health-related data

Sociodemographic and health-related data were collected using a questionnaire developed for this study, including age, sex, degree program, and living arrangement (with parents; university residence; shared student apartment). Health-related variables included blood pressure, oxygen saturation, resting heart rate, and self-reported tobacco and alcohol consumption.

Blood pressure was measured using a manual sphygmomanometer (Boso Manuell, 0–300 mmHg; Boso, Germany). Systolic blood pressure (SBP) was defined as Korotkoff phase I and diastolic blood pressure (DBP) as Korotkoff phase V (27). The recorded value corresponded to the mean of at least three measurements taken at 5-min intervals. Values were interpreted according to recommendations of the European Societies of Hypertension and Cardiology (28).

Oxygen saturation and heart rate were measured using a pulse oximeter (Tuffsat, Datex-Ohmeda, GE Healthcare, Madrid, Spain) (29, 30). Prior to measurement, participants’ fingertips were warmed/massaged to avoid cold-induced perfusion artifacts, and the sensor was placed on the fingertip following manufacturer instructions.

### 2.4 Physical activity and energy expenditure data

Physical activity was assessed using a validated 24-h activity questionnaire (31), and total energy expenditure was estimated using Institute of Medicine (IoM) equations (32). The activity questionnaire recorded the time (minutes) spent in daily activities to derive an individual coefficient for each participant. Time spent in each activity was multiplied by a different intensity factor according to the guidelines of the World Health Organization guidance (33), and the resulting coefficient was mapped to the corresponding IoM physical activity level category for energy expenditure estimation. From the 24-h activity questionnaire, an individual physical activity coefficient (IPAC), reflecting overall activity level. IPAC was used as a continuous covariate in the adjusted regression models.

### 2.5 Anthropometry and body composition

Anthropometric measurements were obtained according to ISAK guidelines (34) with participants barefoot and wearing light clothing. Body weight was measured using a digital scale (Seca Alpha; 0.1–150 kg; precision 0.1 kg; Seca, Hamburg, Germany). Height was measured with a stadiometer (Harpenden; 70–205 cm; precision 0.1 cm; Holtain Ltd., Crymych, United Kingdom). Body mass index (BMI) was calculated as weight (kg)/height^2^ (m^2^) and participants were classified as underweight, normal weight, overweight, or obese according to standard criteria (35).

Waist and hip circumferences were measured with an inextensible tape (Holtain; 0–150 cm; precision 0.1 cm) and expressed in centimeters. Skinfold thicknesses (biceps, triceps, and subscapular) were measured on the non-dominant side using a Holtain skinfold caliper (constant pressure 10 g/mm^2^; 0–40 mm; precision 0.1 mm). Body composition was assessed by single-frequency bioelectrical impedance analysis (OMRON BF306 Body Fat Monitor; OMRON, Madrid, Spain). Waist-to-hip ratio, waist-to-height ratio, body fat percentage, and fat-free mass were derived as described elsewhere (36).

### 2.6 Dietary assessment

Dietary intake was assessed using a 4-day food and beverage record (including one Sunday/holiday) based on the method described by Ortega et al. (37). Selenium intake was estimated using foods reported in the 4-day dietary record. Each item was entered into the DIAL software (38) which uses the Spanish Food Composition Database of Ortega et al. (39) as its primary nutrient source. Total selenium intake was calculated as the sum of selenium contributed by all consumed items, based on their reported portion sizes of each category, which strengthens the accuracy of nutrient estimation in comparison with approaches based on group-level averages. Participants recorded all foods and beverages consumed inside and outside the home using kitchen scales and/or household measures, following standardized instructions provided by the research team. Dietary data were analyzed using DIAL software (38). Energy intake and selenium intake were estimated, and compliance with recommended intakes was assessed using Spanish reference values for this age group (40).

To evaluate the plausibility of reported energy intake, each participant’s energy intake (EI) was compared with estimated total energy expenditure (EE), calculated based on age, and sex. The percentage difference between EI and EE was calculated as:


Percentage difference(%)=(EE−EI)/EE×100.

Positive values indicate EI < EE (potential under-reporting), whereas negative values indicate EI > EE (potential over-reporting) (41).

### 2.7 Blood sampling and selenium determination

Fasting blood samples were collected in the morning after a 10–12 h overnight fast by venipuncture of the cubital vein. EDTA tubes were used for hematological analyses, and trace-element–free tubes were used for selenium determination. Serum selenium concentration was measured by inductively coupled plasma mass spectrometry (ICP-MS). Serum samples were digested with nitric acid (HNO₃) and hydrogen peroxide (H₂O₂) and subsequently analyzed by ICP-MS, where selenium ions were separated by mass-to-charge ratio and quantified as previously described (42). Serum selenium was additionally categorized according to a functional adequacy threshold. Participants were classified as being below the lower reference value when serum selenium was <80 μg/L, based on the range proposed by Thomson, who concludes that 80–95 μg/L (1.0–1.2 μmol/L) is sufficient for maximization of plasma GPx and selenoproteins P (43).

### 2.8 Cognitive assessment

Cognitive function was assessed using the Wechsler Adult Intelligence Scale-Fourth Edition (WAIS-IV) (44) which includes 10 core subtests and 5 supplemental subtests. When a core subtest was invalid, the corresponding supplemental subtest was administered according to WAIS-IV guidelines. Four index scores were derived from core subtests: Verbal Comprehension Index (VCI), Perceptual Reasoning Index (PRI), Working Memory Index (WMI), and Processing Speed Index (PSI).

The VCI is based on Similarities, Vocabulary, and Information (with Comprehension as supplemental) and reflects verbal reasoning and concept formation. The PRI is based on Block Design, Matrix Reasoning, and Visual Puzzles (with Figure Weights and Picture Completion as supplemental) and reflects non-verbal reasoning and perceptual organization. The WMI is based on Digit Span and Arithmetic (with Letter-Number Sequencing as supplemental) and reflects attention and working memory processes. The PSI is based on Symbol Search and Coding (with Cancelation as supplemental) and reflects the speed and efficiency of visual information processing and graphomotor speed.

Raw subtest scores were converted to age-corrected scaled scores and then to composite index scores (mean 100, SD 15) using normative conversion tables (44). Global intellectual performance was summarized using the TIQ, derived from the sum of scaled scores contributing to the four index scores, and considered a robust measure of general cognitive ability across multiple domains (45).

For descriptive analyses, participants were classified into two groups using an operational cut-off: higher cognitive performance (TIQ ≥ 90) and lower cognitive performance (TIQ < 90). Index scores (VCI, PRI, WMI, PSI) were similarly categorized as ≥90 vs. <90. In addition, cognitive indices were stratified in quartiles and mean serum selenium or selenium intake were calculated for each group ≥90 vs. <90.

### 2.9 Statistical analysis

Dietary data were tabulated using DIAL software (for Windows, version 3.0.0.5) (38). All other socio-health, anthropometric, hematological, biochemical and cognitive capacity data were entered into the same database. Data analysis began with a descriptive analysis of absolute and relative frequencies, median, means and standard deviations. Statistical analyses included Student’s *t*-test, the chi-square, Mann Whitney test and Kruskal–Wallis test. Differences between mean values were considered statistically significant at *p* < 0.05. D’Agostino and Pearson’s test was used to check whether the values followed a normal distribution. Spearman’s correlation analysis was used to assess the correlation between data variables from different experimental groups. Primary analysis: associations of dietary selenium intake (μg/day) and serum selenium concentration (μg/L) with global intelligence (TIQ) were evaluated using linear regression models. Secondary analyses examined associations with WAIS-IV index scores (VCI, PRI, WMI, PSI). Models were fitted unadjusted and adjusted for prespecified covariates (age, sex, IPAC, BMI, and total energy intake). Regression assumptions were evaluated (linearity, residual normality and homoscedasticity), and collinearity was assessed (variance inflation factor). Results are reported as *β* coefficients with 95% confidence intervals. Given the possibility of non-linear associations between selenium status and cognitive outcomes, scatterplots with LOWESS (Locally Weighted Scatterplot Smoothing) and spline smoothing were also inspected to evaluate potential non-linear patterns. All statistical analyses were performed using GraphPad Prism 8.0 software (GraphPad Software, San Diego, CA, United States).

## 3 Results

A total of 143 students were initially recruited (79 female, 64 male). Eleven were excluded due to incomplete data and/or exclusion criteria, leaving 132 university students for analysis (71 female, 53.7%; 61 male, 46.2%).

Baseline characteristics by sex are presented in Table 1. Male showed higher systolic and diastolic blood pressure than female (both *p* < 0.001), although no participant met criteria for hypertension.

**Table 1:** Sociodemographic, clinical, physical activity, and anthropometric characteristics of participants by sex.

Data	Female	Male
Personal and lifestyle data		
Age (years)	19 (18–21)	21 (19–23) ***
Place of cohabitation (%)		
Living with parents	13.4	29.5
Student flat	71.6	52.5
Student residence	14.9	18
Smoking habit (%)		
Non-smoker	67.6	78.7
Ex-smoker	12.5	10.4
Smoker	32.4	21.3
Cigarettes/day	6.0 ± 4.8	6.6 ± 4.9
Alcohol consumption (g/day)	4.1 ± 9.2	5.5 ± 13.1
Health data		
Systolic blood pressure (mmHg) (normal values) ref. (86) Optimal systolic (%) Normal systolic (%) High-normal systolic (%) Hypertension (%)	96.5 ± 11.3 (120–129) 94.4 5.6 0.0 0.0	108.4 ± 10.6 *** (120–129) 62.3 37.7 0.0 0.0
Diastolic blood pressure (mmHg) (normal values) ref. (47)	60.7 ± 10.8 (80–84)	68.7 ± 9.7 *** (80–84)
Optimal diastolic (%)	94.4	72.1
Normal diastolic (%)	5.6	27.9
High-normal diastolic (%)	0.0	0.0
Hypertension (%)	0.0	0.0
Oxygen saturation (%) (normal values) ref. (30)	97.2 ± 1.5 (>95)	96.4 ± 4.6 (>95)
Heart rate (beats/min) (normal values) ref. (87)	86 (74–95) (60–90)	80 (70.5–85.5) * (60–90)
Low (%)	1.4	3.3
Normal (%)	85.9	91.8
High (%)	12.7	4.9
Anthropometric data		
Weight (kg)	58.8 (53–64.7)	77.2 (68.1–81.5) ****
Height (cm)	161 (158–166)	175 (172–180) ****
BMI (kg/m^2^)	22.3 (20.4–23.8)	24.8 (22.2–27) ****
Weight status (%) ref. (35)		
Underweight (BMI < 18.5)	4.2	0.0
Normal weight (BMI: 18.5–24.9)	77.5	54.1
Overweight (BMI: 25.0–29.9)	16.9	34.4
Obese (BMI ≥ 30.0)	1.4	11.5
Waist circumference (cm)	74 (69.5–80.5)	87 (81.7–94.5) ****
Hip circumference (cm)	96 ± 13.9	100 ± 8.2 *
Waist to hip ratio	0.78 (0.74–0.82)	0.88 (0.85–0.91) ****
Waist to height ratio	0.46 (0.42–0.51)	0.5 (0.46–0.53) ***
Bicipital skinfold (mm)	10 (6–13)	7 (5.5–9.5) **
Tricipital skinfold (mm)	15 (11–20)	11 (8–15.5) **
Subscapular skinfold (mm)	14.5 (11–22)	15 (11–21)
Body fat (%)	32.3 ± 5.4	23.3 ± 7.2
Fat free mass (%)	68.8 (63.7–72.3)	76 (71.6–82.3) ****
Physical activity data		
Sports practice (hours/day)	0.21 ± 0.36	0.77 ± 0.59
Individual Physical Activity Coefficient (IPAC)	1.35 (1.32–1.38)	1.40 (1.36–1.48) ****

Data are means ± standard deviation or median (IQR) of female (*n* = 71) and male (*n* = 61) participants. *p < 0.05, **p < 0.01, ***p < 0.001, and ****p < 0.0001 significantly different from the corresponding female value according to Students’ *t*-test or Mann–Whitney test for variables following a normal or not normal distribution, respectively BMI, body mass index.

Male also exhibited higher body weight and height, waist and hip circumferences, BMI, and fat-free mass (Table 1). Overweight/obesity was more frequent in male (34.4 and 11.5%, respectively), whereas 4.2% of female were underweight.

Regarding physical activity, most students have a sedentary lifestyle with little sports participation, particularly among women (1.51 ± 2.55 h/week) (Table 1), which does not meet WHO guidelines, as they perform less than 150 min of moderate aerobic activity per week (or the equivalent in vigorous activity) (46).

Furthermore, the individual physical activity coefficient (IPAC), which is higher in male participants 1.40 (1.36–1.48) vs. 1.35 (1.32–1.38) (*p* < 0.0001) (Table 1), indicates that the university student population studied has an activity level classified as low (1.4–<1.6).

Energy intake was higher in male than in female. When analyzing the relationship between declared energy intake and theoretical energy expenditure, a mean tendency toward underestimation was observed (1.6%) (Table 2), meaning that the declared intake is less than the total estimated energy expenditure. Mean dietary selenium intake in the overall sample was 96.6 (78.1–122.8) μg/day, with higher intake in male compared with female. A small proportion of female had selenium intakes below 100% of the recommended intake and none of the male had low intakes of the mineral. Serum selenium concentration was 85.5 (73.8–103.3) μg/L, and it was higher in male than female. Taking as a cutoff point 80–95 μg/L indicated by Thomson et al. (43) as the level necessary to achieve the maximum activity of GPx, selenoprotein P and other selenoproteins, one third of the university population showed inadequate levels, mainly the female group (Table 2).

**Table 2:** Energy and selenium intake, and selenium status.

Data	Total	Female	Male
Energy (kcal/day)	2031 (1739–2,539)	1835 (1667–2020)	2,502 (2129–2,907)****
Energy contribution RI (%)	94.5 (85.8–107.9)	94.7 (90.9–104.3)	93.4 (80.3–115)
< 100% RI (%)	62.1	64.8	59.0
< 67% RI (%)	6.1	2.8	9.8
Energy Expenditure (kcal/day)	2,185 (1880–2,636)	1896 (1775–2033)	2,649 (2547–2,813) ****
Under-reporting (%)	1.6	0.4	3.0
Selenium intake (μg/day)	96.6 (78.1–122.8)	82.7 (72.9–104)	108 (93.9–134.5) ****
Se contribution RI (%)	161 (137.5–213.5)	159.6 (134–204)	172.9 (137.6–228.6)
< 100% RI (%)	3.7	7.0	0.0
< 67% RI (%)	0.7	1.4	0.0
Biochemistry			
Serum selenium (μg/L) (Reference Range 80–95 μg/L) (43)	85.5 (73.8–103.3)	78 (68–86)	103.2 (91.5–115.9)****
Serum selenium (% below Reference Range)	36.4	53.5	16.4

Differences based on sex (mean ± SD or %).

The reference range used to classify serum selenium adequacy (<80 μg/L) is based on functional thresholds for selenoprotein optimization (43). However, this cut-off does not account for sex-specific physiological differences. Spanish Agency for Food Safety and Nutrition (AESAN) (47) recommends higher dietary selenium intakes for male (70 μg/day) than for female (55 μg/day), suggesting that serum requirements may also require sex-specific interpretation. These differences may partly explain the higher proportion of female classified as having low serum selenium in our study.

Data are median (IQR) or %. **** *p* < 0.0001 vs. the corresponding female value according to Mann–Whitney test for variables following a not normal distribution. RI, recommended intake. Under-reporting (%) was derived from the EI–EE comparison (as defined in Methods). Se: selenium. Serum selenium (% below Reference Range): < 80 μg/L.

**Figure 1:**
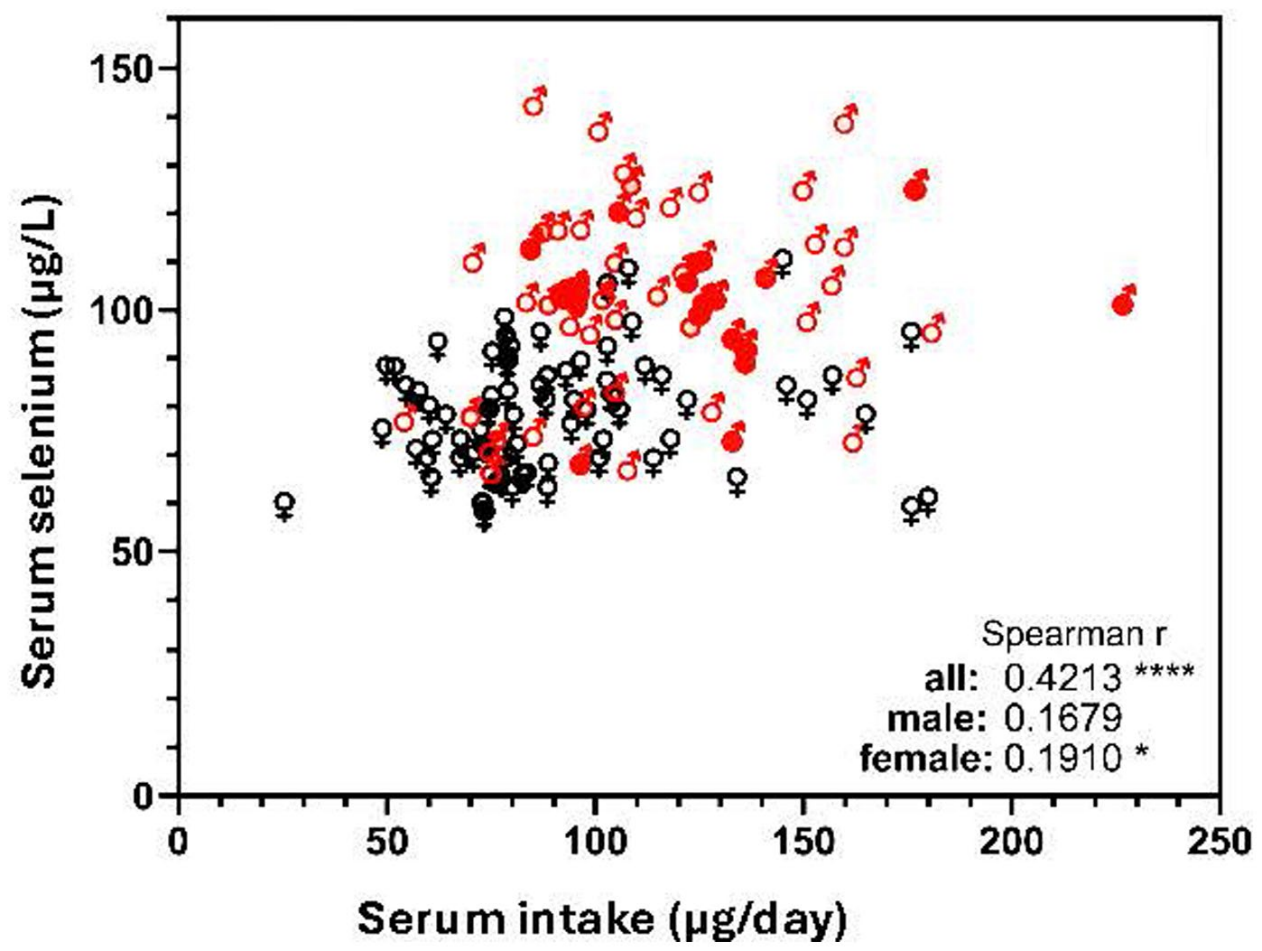
Association between dietary selenium intake (μg/day) and serum selenium concentration (μg/L) in the overall sample and stratified by sex (Spearman’s *r*). Each point represents one participant. ****p < 0.0001; *p < 0.05. **♀**: Female; ♂: male.

Sex differences in selenium intake were mirrored by differences in the consumption of major selenium-contributing food groups (Table 3), including cereals/pulses, vegetables, meat–fish–eggs, and dairy products (all *p* < 0.001). Although selenium intake was estimated using item-specific composition data, variability in selenium content due to soil differences or food origin cannot be fully captured by composition tables. Notably, female students reported lower intakes of meat, fish, and eggs and cereals and pulses than males (*p* < 0.001). These food groups represented the main sources of selenium in most diets; however, selenium content may vary depending on the origin of feed ingredients used in animal production and on soil selenium availability for crop cultivation. Serum selenium concentrations were also higher in male than in female, and although mean values were within the reference range, 53.5% of female and 16.4% of male presented values below reference range (Table 2). Dietary selenium intake was positively correlated with serum selenium concentration in the overall sample (Spearman’s *r* = 0.4213, *p* < 0.0001) and in female (Spearman’s *r* = 0.1910, *p* < 0.05) (Figure 1).

**Table 3:** Food consumption (servings/day) among young university students.

Food group	Female	Male
Cereals and pulses	3.9 (3.2–4.6)	5.3 (3.8–7.0) ****
Vegetables	2.1 ± 1.1	3.1 ± 1.5***
Fruits and derivatives	1.1 ± 1.2	1.5 ± 1.1
Meat, fish and eggs	2.6 ± 1.1	3.5 ± 1.4***
Dairy products	1.5 (0.9–2.1)	2.1 (1.5–2.8)****

Differences based on sex. Data are mean ± standard deviation (SD) or median (IQR). ***p < 0.001, ****p < 0.0001 vs. the corresponding female value according to Student t test or Mann–Whitney test for variables following a normal or not normal distribution, respectively.

**Figure 2:**
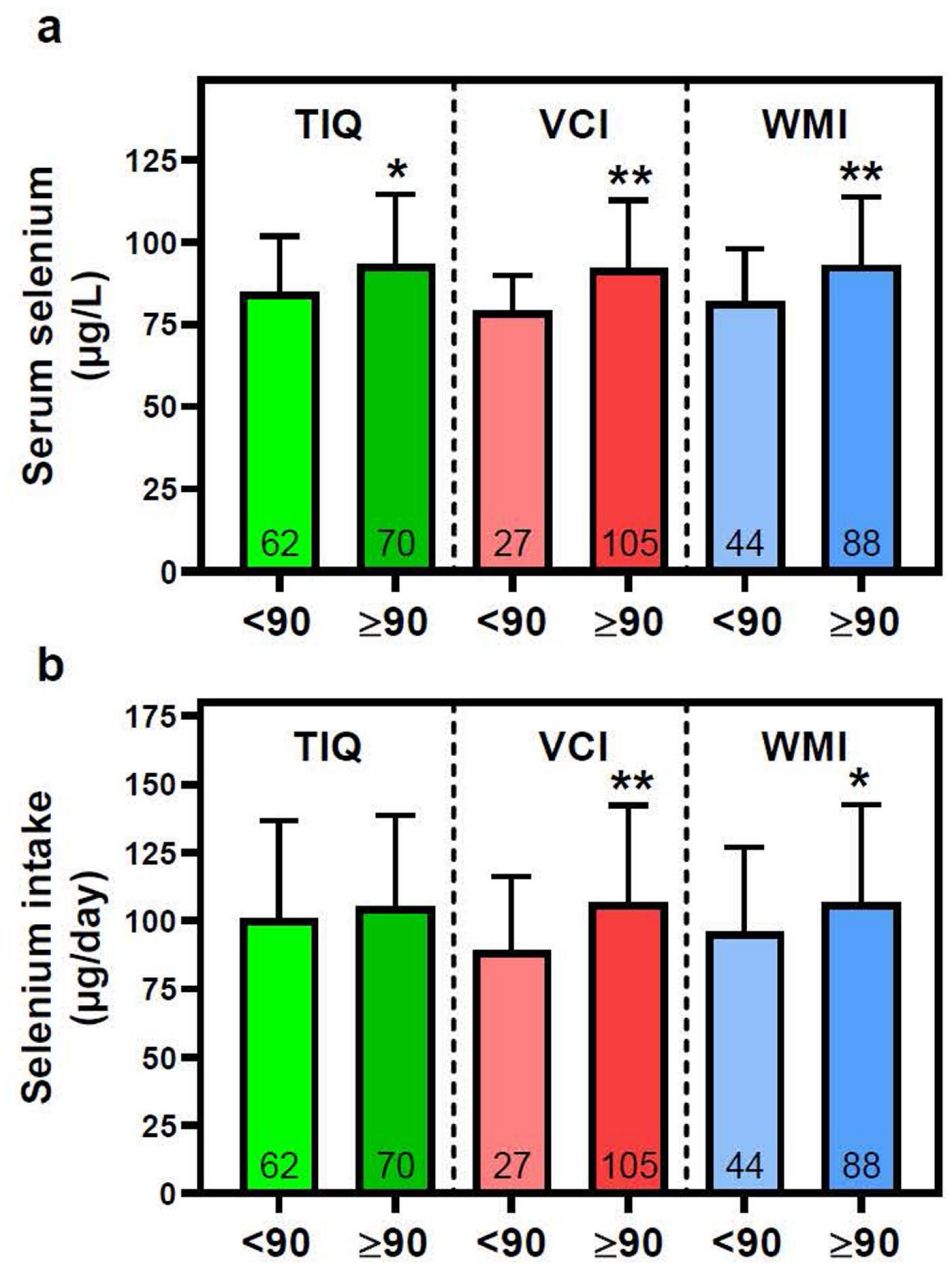
Serum selenium concentration **(a)** and dietary selenium intake **(b)** according to cognitive performance categories (TIQ, VCI, and WMI; <90 vs. ≥90). Bars represent mean ± SD; numbers within bars indicate sample size. Group differences were assessed using the Mann–Whitney U test. *p < 0.05, p** < 0.01.

When intellectual ability was examined according to selenium status, serum selenium concentrations were lower in students with low-average intelligence (TIQ < 90) than in those with medium-high intelligence (TIQ ≥ 90) (*p* < 0.05) (Figure 2). In addition, prevalence of below reference values of serum selenium was more frequent in the TIQ < 90 group (Table 4).

**Table 4:** Energy intake, dietary selenium intake, and serum selenium concentration by sex and TIQ category (TIQ < 90 vs. TIQ ≥ 90).

Data	Total		Female		Male	
	TIQ < 90 (*n* = 62)	TIQ ≥ 90 (*n* = 70)	TIQ < 90 (*n* = 45)	TIQ ≥ 90 (*n* = 26)	TIQ < 90 (*n* = 17)	TIQ ≥ 90 (*n* = 44)
Energy (kcal/day)	1900 (1694–2,255)	2,298 (1807–2,678) ††	1835 (1667–1999)	1833 (1671–2,237)	2,316 (2032–2,877) ****	2,561 (2166–3,055) ****
Energy contribution RI (%)	94.2 (85.9–104.5)	97.2 (83.6–111.3)	94.7 (90.9–104.2)	97.8 (90.5–105.7)	84.1 (75.6–108.8)	96.2 (82.6–117.4)
Selenium intake (μg/day)	91 (75.1–119)	100 (82.4–126)	83.3 (71.7–105)	82.0 (73.2–104.3)	118 (90.7–133) **	106 (93.7–139.8) ***
Se contribution RI (%)	161 (132.7–215)	160.6 (138.2–215.1)	161 (131.3–207)	158.9 (139.3–204.5)	168.6 (129.6–239.3)	174.3 (138.1–227.5)
< 100% RI (%)	3.2	4.3	4.4	11.5	0.0	0.0
< 67% RI (%)	0.0	1.4	0.0	3.8	0.0	0.0
Biochemistry						
Serum selenium (μg/L)	81.5 (72–94.3)	93.5 (74–107.1) †	79 (69.5–86.5)	74 (66.5–85.5)	100.9 ± 18.1 ***	103.3 ± 19.1 ***
Serum selenium (% below reference range)	43.5	30.0	51.1	57.7	23.5	13.6

Data are mean ± SD, median (IQR) or %. †*p* < 0.05 and ††*p* < 0.01 for TIQ ≥ 90 vs. TIQ < 90 (total sample). *p < 0.05 and ***p < 0.001 for males vs. females within the same TIQ category according to Student t test or Mann–Whitney test for variables following a normal or not normal distribution, respectively. RI, recommended intake. Se, selenium. Serum selenium (% below reference range): <80 μg/L.

Dietary selenium intake also tended to be lower among participants with TIQ < 90, although this difference did not reach statistical significance (Table 4). No statistically significant differences were observed in sex-stratified comparisons (Table 4).

In addition to TIQ, selenium intake and status also differed across specific cognitive indices (Figure 2). Participants with low-average Verbal Comprehension Index (VCI < 90) showed lower dietary selenium intake and lower serum selenium concentrations compared with those with VCI ≥ 90 (*p* < 0.01). Similarly, serum selenium concentrations and dietary selenium intake were lower in students with low-average Working Memory Index (WMI < 90) than in those with WMI ≥ 90 (*p* < 0.05 and *p* < 0,01, respectively).

**Figure 3:**
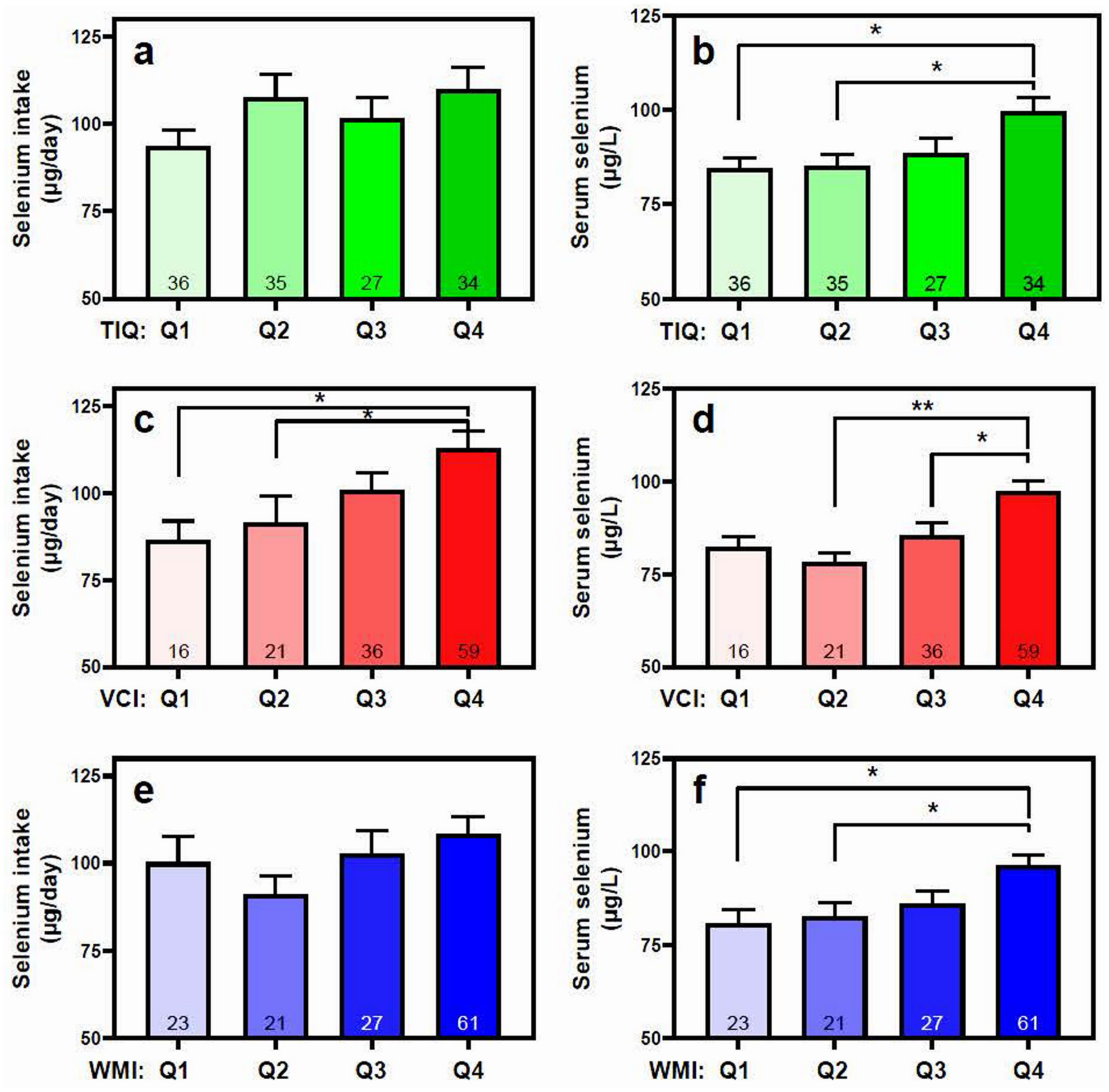
Dietary selenium intake **(a,c,e)** and serum selenium concentration **(b,d,f)** according to cognitive performance categories (TIQ, VCI, and WMI) stratified in quartiles (Q1–Q4). Bars represent mean ± SEM; numbers within bars indicate sample size. Group differences were assessed with Kruskal–Wallis tests, followed when appropriate by Dunn’s multiple-comparisons test. Significant pairwise differences are indicated by brackets (*p < 0.05, ** *p* < 0.01).

In addition, cognitive indices were stratified in quartiles and mean serum selenium or selenium intake were calculated for each group (Figure 3). A Kruskal–Wallis test showed a significant difference in mean serum selenium value between the TIQ quartiles [*H*_(3, *N* = 132)_ = 10.75, *p* = 0.013]. *Post-hoc* pairwise comparisons using Dunn’s test indicated that the highest TIQ quartile (Q4) differed significantly from the first (Q1) and second (Q2) quartiles (adjusted *p* = 0.026 and 0.028, respectively), whereas no other pairwise comparisons were statistically significant (all adjusted *p* > 0.28). However, no statistically significant differences were found in selenium intake across the four TIQ quartile groups [*H*_(3, *N* = 132) =_ 5.78, *p* = 0.12].

For VCI quartiles, a significant difference in mean serum selenium value between the VIC quartiles was found [*H*_(3, *N* = 132)_ = 17.22, *p* = 0.0006]. *Post-hoc* test indicated that the VIC Q4 quartile differed significantly from the Q2 and Q3 quartiles (adjusted *p* = 0.0020 and 0.0410, respectively). A significant difference was also found in mean selenium intake value between the VIC quartiles [*H*_(3, *N* = 132)_ = 12.37, *p* = 0.0062], whereas *post-hoc* test revealed that VIC Q4 differed significantly from the Q1 and Q2 quartiles (adjusted *p* = 0.0327 and 0.0387, respectively).

Finally, for WMI quartiles, while no significant differences were found concerning selenium intake, the mean serum selenium differed across WMI quartiles [*H*_(3, *N* = 132)_ = 13.64, *p* = 0.0034] with significant differences between WMI Q4 and Q1 (adjusted *p* = 0.0181) or Q2 (adjusted *p* = 0.0411).

**Figure 4:**
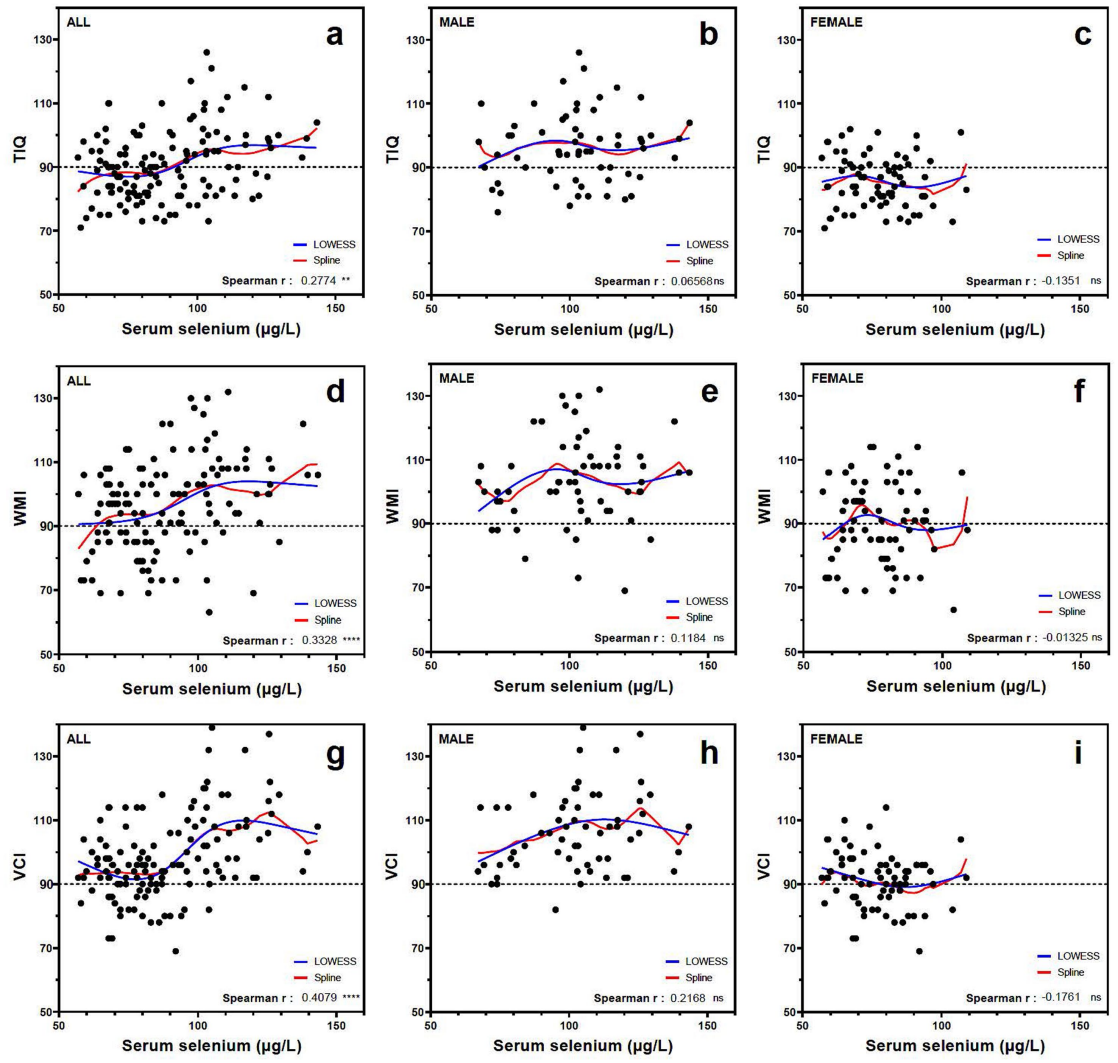
Relationship between cognitive indices and serum selenium concentration (μg/L) in the overall sample and stratified by sex. Each dot represents one participant. TIQ **(a–c)**, WMI **(d–f)**, and VCI **(g–i)**. Dashed lines indicate the cut-off value of 90 points. Spearman’s *r* and *p*-values (*p < 0.05, **p < 0.01, and ****p < 0.0001) are shown in each panel. Curves were fitted using LOWESS (blue) and spline regression (red).

**Figure 5:**
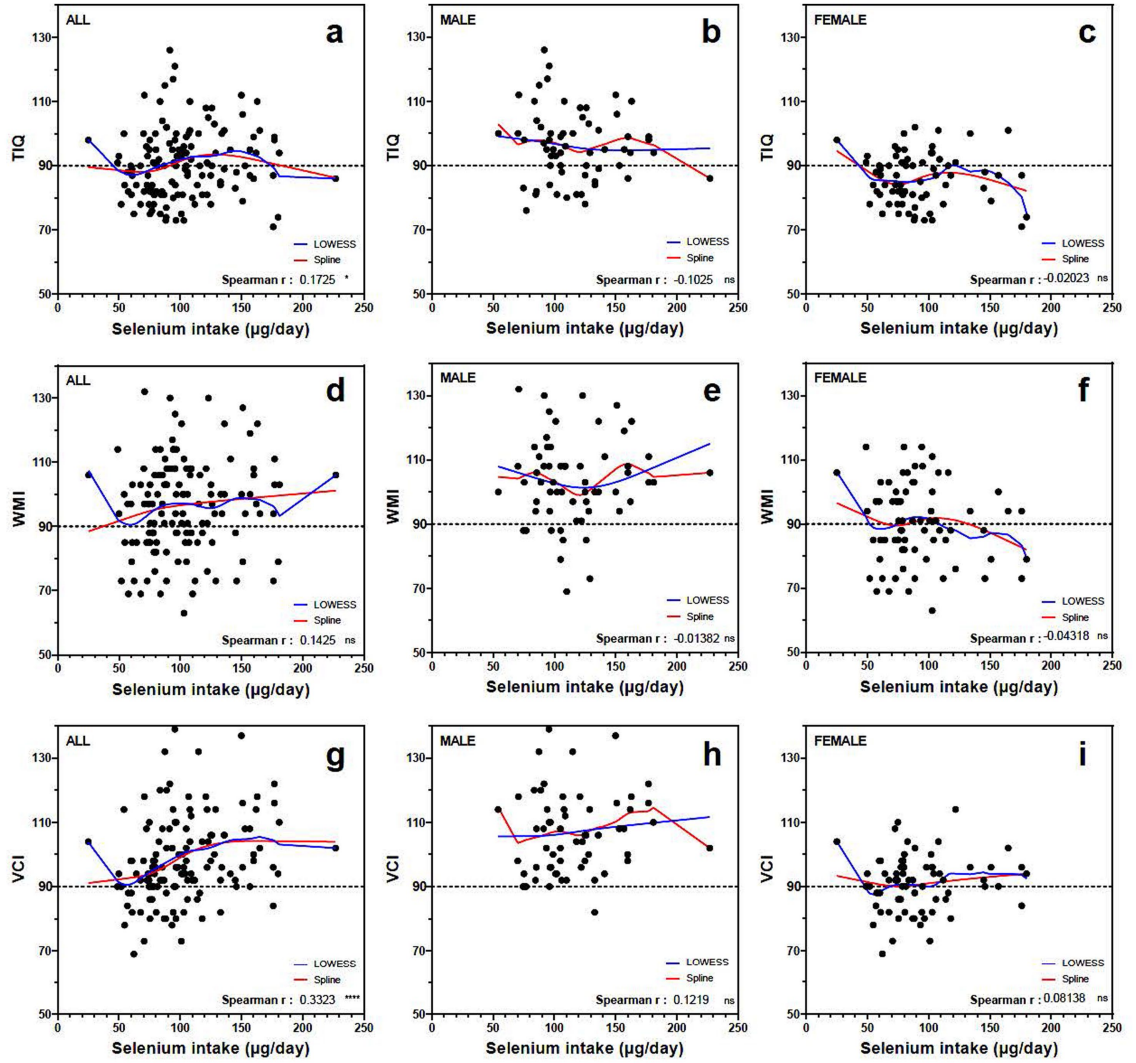
Relationship between cognitive indices and selenium intake (μg/day) in the overall sample and stratified by sex. Each dot represents one participant. TIQ **(a–c)**, WMI **(d–f)**, and VCI **(g–i)**. Dashed lines indicate the cut-off value of 90 points. Spearman’s *r* and *p*-values (*p < 0.05, **p < 0.01, and ****p < 0.0001) are shown in each panel. Curves were fitted using LOWESS (blue) and spline regression (red).

Figures 4, 5 shows the associations between selenium status and cognitive indices in the overall sample and stratified by sex. Serum selenium (Figure 4) was positively correlated with TIQ, WMI and VCI, with the strongest association observed for VCI. Specifically, the Spearman correlation coefficients were *r* = 0.2774 for TIQ, *r* = 0.3328 for WMI, and *r* = 0.4079 for VCI, all statistically significant. In contrast, selenium intake (Figure 5) showed weaker associations with cognitive indices: a modest positive correlation with TIQ (*r* = 0.1725, *p* < 0.05), no significant association with WMI (*r* = 0.1425, not significant), and a significant positive correlation with VCI (*r* = 0.3323, *p* < 0.001). Sex-stratified analyses indicated that serum selenium showed a more consistent positive relationship with cognitive outcomes in men, whereas no significant associations were found in women. For dietary selenium intake, only VCI remained positively associated in men, and no significant associations were observed in women (Table 5).

**Table 5:** Energy intake, dietary selenium intake, and serum selenium concentration by sex and cognitive abilities (VCI, WMI, PRI y PSI) category (cognitive abilities < 90 vs. ≥ 90).

Total	Verbal comprehension index (VCI)		Working memory index (WMI)		Perceptual reasoning index (PRI)		Processing speed index (PSI)	
	<90 (*n* = 27)	≥90 (*n* = 105)	<90 (*n* = 44)	≥90 (*n* = 88)	<90 (*n* = 67)	≥90 (*n* = 65)	<90 (*n* = 88)	≥90 (*n* = 44)
Energy (kcal/day)	1806 (1667–2020)	2,153 (1820–2,632) †††	1840 (1673–2035)	2,255 (1813–2,742) ††††	1952 (1682–2,304)	2,257 (1833–2,689) ††	2022 (1808–2,571)	2040 (1679–2,427)
Energy contribution RI (%)	93.5 (90.9–97.9)	96.8 (82.8–108.3)	94.5 (85.8–104.2)	94.5 (84.5–110)	93.4 (85.1–103)	97.8 (88.1–113.2)	95.1 (84.3–107.9)	94.2 (87.4–107.8)
Selenium intake (μg/day)	88.2 (67.7–103)	101 (79.5–127) ††	88.6 (74.6–111.5)	97.9 (82.9–127.5) †	98.1 (77.1–122)	96.1 (80–124.5)	95.9 (79.1–124.5)	102.5 (75.3–117)
Se contribution RI (%)	161.3 (123.1–193.2)	158.2 (138.1–224.1)	157 (133.7–207)	169.1 (137.5–222.9)	168.6 (143.1–212)	158.2 (131.1–221.3)	157.7 (137–222.6)	181.6 (141.7–211.5)
<100 IR (%)	3.7	3.8	4.5	3.4	3.0	4.6	3.4	4.5
<67 IR (%)	0.0	0.9	0.0	1.1	0.0	1.5	1.1	0.0
Serum selenium (μg/L)	79 (70–86)	91 (74–106.3) ††	80.5 (72–87.7)	93 (74.2–106.9) ††	84 (72–104)	87.1 (74–102.9)	89 (75–104.1)	81.5 (68.2–97.5) †
Serum selenium (% below reference value)	51.9	32.4	45.5	31.8	38.8	33.8	34.1	40.9
Female	<90 (*n* = 26)	≥90 (*n* = 45)	<90 (*n* = 35)	≥90 (*n* = 36)	<90 (*n* = 41)	≥90 (*n* = 30)	<90 (*n* = 41)	≥90 (*n* = 30)
Energy (kcal/day)	1795 (1667–2007)	1843 (1669–2040)	1819 (1666–1981)	1843 (1687–2,190)	1784 (1664–1971)	1842 (1698–2,161)	1835 (1646–1970)	1833 (1673–2,273)
Energy contribution RI (%)	93.5 (90.3–96.3)	98.3 (91.1–105.2)	94.7 (88.4–104.2)	96.2 (92–107)	94.3 (90–103)	97.8 (90.9–107.2)	94.7 (90.8–102.5)	95.2 (90.7–109.3)
Selenium intake (μg/day)	87.5 (66.3–101.5)	81.4 (73.5–108.5)	80.5 (72.5–112)	84.8 (73.5–102.8)	88.2 (74.7–103.5)	80.9 (63.8–110.8)	80.5 (70.7–95.8)	97.1 (72.8–110.3)
Se contribution RI (%)	161.2 (122.8–195.4)	156.8 (141.3–206)	156.8 (131.8–224)	160.6 (136.3–203.5)	161.1 (142.5–203)	158.9 (122.8–212.5)	154.2 (131.3–191)	178.5 (139.3–216)
<100 RI (%)	3.8	8.9	5.7	8.3	4.9	10.0	7.3	6.7
<67 IR (%)	0.0	2.2	0.0	2.7	0.0	3.3	2.4	0.0
Serum selenium (μg/L)	78.5 (69.7–85.2)	78 (66–87)	79 (67–85)	76 (68.2–86.7)	78 (68–86.5)	77 (67.7–85.5)	78 (68.5–86)	77.5 (68–86.2)
Serum selenium (% below reference value)	53.8	53.3	51.4	55.6	53.7	53.3	53.7	53.3
Male	<90 (*n* = 1)	≥90 (*n* = 60)	<90 (*n* = 9)	≥90 (*n* = 52)	<90 (*n* = 26)	≥90 (*n* = 35)	<90 (*n* = 47)	≥90 (*n* = 14)
Energy (kcal/day)	3,067 (3067–3,067)	2,542 (2121–2,904) ****	2,452 (1913–2,623) ***	2,536 (2166–2,993) ****	2,290 (2009–2,800) ****	2,630 (2239–3,067) ****	2,530 (2147–3,067) ****	2,354 (2007–2,790) *
Energy contribution RI (%)	116.3 (116.3–116.3)	93.2 (80.2–113.4)	86.1 (73.9–116.2)	93.8 (81.7–115.6)	86.3 (73.1–103.8)	98.8 (83.2–117.5)	95.7 (80.4–119.6)	92.9 (73.4–102.3)
Selenium intake (μg/day)	133 (133–133)	107.5 (93.7–135.3) ***	105 (87.2–118)	112 (93.7–139.8) ****	114 (95.2–154) ***	105 (91.6–133) ***	110 (93.6–136) ****	106.5 (93.5–128.3)
Se contribution RI (%)	190 (190–190)	170.7 (137.5–228.6)	157.1 (124.6–197.1)	173.6 (137.5–245)	177.1 (143.6–233.9)	154.3 (136.7–228.6)	168.6 (137.3–230)	181.6 (140.4–211)
<100 RI (%)	0.0	0.0	0.0	0.0	0.0	0.0	0.0	0.0
<67 IR (%)	0.0	0.0	0.0	0.0	0.0	0.0	0.0	0.0
Serum selenium (μg/L)	95.1 (95.1–95.1)	103.2 (90.7–116.4) ****	99 (77.5–111.6) *	103.6 (95.3–116.4) ****	106.3 (95.4–120.3) ****	102.4 (87.1–110.8) ****	103 (95.1–117.4) ****	103.3 (83–110.9) ***
Serum selenium (% below Reference Range)	0.0	16.7	22.2	15.4	15.4	17.1	17.0	14.3

Data are median (IQR) or %. †*p* < 0.05, ††*p* < 0.01, †††*p* < 0.001, and ††††*p* < 0.0001 for VCI, WMI, PRI and PSI ≥ 90 vs VCI, WMI, PRI and PSI < 90 (total sample). *p < 0.05, **p < 0.01, ***p < 0.001, and ****p < 0.0001 for males vs. females within the same VCI, WMI, PRI and PSI category, according to Mann–Whitney test. RI, recommended intake. Se, selenium. Serum selenium (% below reference range): <80 μg/L.

Because dietary selenium intake and serum selenium were moderately correlated, both variables were not included in the same multivariable model to avoid multicollinearity. Instead, separate regression models were fitted for each selenium measure. Therefore, hierarchical multiple linear regression was used to examine the association between cognitive indices and dietary selenium intake (Table 6) or serum selenium (Table 7). In the fully adjusted model with TIQ as the dependent variable (Table 6), male sex was independently associated with higher TIQ (*β* = 9.95; 95% CI: 5.57–14.33; *p* < 0.001), as was higher energy intake (*β* = 0.0049 TIQ points per kcal/day; 95% CI: 0.00055–0.00927; *p* = 0.028). Dietary selenium intake was inversely associated with TIQ (*β* = −0.067 TIQ points per μg/day; 95% CI: −0.130 to −0.005; *p* = 0.035). The model explained approximately 29% of the variance in TIQ (*R*^2^ = 0.29). Age and BMI were not significant predictors (Table 6).

**Table 6:** Multiple linear regression models for associations between selenium intake and cognitive outcomes.

Outcome	Units	β_0_	β_1_	β_2_	β_3_	β_4_	β_5_	β_6_
		Not applicable	Categorical	Per year	Per kcal/day	Per kg/m^2^		Per μg/day
		Intercept	Sex	Age	Energy	BMI	IPAC	Selenium intake
TIQ	Unstandardized estimate	96.71	9.954	−0.7084	0.00491	0.005686	−1.302	−0.06752
*R*^2^ = 0.2894	95% CI	77.19 to 116.2	5.574 to 14.33	−1.470 to 0.05291	0.0005459 to 0.009274	−0.4907 to 0.5021	−5.144 to 2.539	−0.1301 to −0.004953
*n* = 132	Standardized estimate	−4.365E-16	0.4603	−0.1486	0.2603	0.001856	−0.05115	−0.2161
	95% CI	−0.1487 to 0.1487	0.2578 to 0.6629	−0.3083 to 0.01110	0.02895 to 0.4917	−0.1602 to 0.1639	−0.2020 to 0.09972	−0.4163 to −0.01585
	*p*-value	*<0.0001*	*<0.0001*	*0.0679*	*0.0278*	*0.982*	*0.5035*	*0.0346*
VCI	Unstandardized estimate	99.69	15.56	−0.5343	0.002482	−0.1458	−2.371	0.008191
*R*^2^ = 0.3796	95% CI	76.90 to 122.5	10.45 to 20.68	−1.423 to 0.3548	−0.002614 to 0.007579	−0.7256 to 0.4339	−6.858 to 2.116	−0.06488 to 0.08127
*n* = 132	Standardized estimate	1.988E-16	0.5758	−0.0897	0.1053	−0.03809	−0.07451	0.02097
	95% CI	−0.1389 to 0.1389	0.3865 to 0.7651	−0.2389 to 0.05955	−0.1109 to 0.3215	−0.1895 to 0.1133	−0.2155 to 0.06647	−0.1661 to 0.2081
	*p*-value	*<0.0001*	*<0.0001*	*0.2365*	*0.337*	*0.6195*	*0.2976*	*0.8248*
WMI	Unstandardized estimate	109.8	13.97	−1.237	0.004036	0.1136	−4.648	−0.05423
*R*^2^ = 0.2777	95% CI	83.49 to 136.1	8.065 to 19.88	−2.264 to −0.2099	−0.001851 to 0.009922	−0.5561 to 0.7832	−9.831 to 0.5349	−0.1386 to 0.03018
*n* = 132	Standardized estimate	−9.983E-17	0.483	−0.1939	0.1599	0.02771	−0.1364	−0.1297
	95% CI	−0.1499 to 0.1499	0.2787 to 0.6872	−0.3550 to −0.03292	−0.07336 to 0.3932	−0.1357 to 0.1911	−0.2885 to 0.01570	−0.3316 to 0.07219
	*p*-value	*<0.0001*	*<0.0001*	*0.0186*	*0.1773*	*0.7377*	*0.0784*	*0.2059*
PRI	Unstandardized estimate	95.95	5.826	−0.6057	0.00682	0.05597	2.335	−0.1292
*R*^2^ = 0.1644	95% CI	71.60 to 120.3	0.3618 to 11.29	−1.555 to 0.3440	0.001376 to 0.01226	−0.5633 to 0.6752	−2.457 to 7.128	−0.2072 to −0.05110
*n* = 132	Standardized estimate	−5.665E-16	0.2342	−0.1105	0.3143	0.01588	0.07972	−0.3593
	95% CI	−0.1612 to 0.1612	0.01455 to 0.4539	−0.2837 to 0.06274	0.06343 to 0.5652	−0.1598 to 0.1916	−0.08389 to 0.2433	−0.5764 to −0.1422
	*p*-value	*<0.0001*	*0.0368*	*0.2092*	*0.0145*	*0.8583*	*0.3367*	*0.0014*
PSI	Unstandardized estimate	83.36	−5.938	0.2185	0.001389	0.02892	−0.3016	−0.03202
*R*^2^ = 0.05182	95% CI	56.87 to 109.9	−11.88 to 0.009651	−0.8151 to 1.252	−0.004535 to 0.007314	−0.6451 to 0.7029	−5.518 to 4.915	−0.1170 to 0.05294
*n* = 132	Standardized estimate	−7.434E-17	−0.2336	0.039	0.06268	0.008033	−0.01008	−0.08717
	95% CI	−0.1717 to 0.1717	−0.4676 to 0.000379	−0.1455 to 0.2235	−0.2046 to 0.3299	−0.1792 to 0.1952	−0.1844 to 0.1642	−0.3185 to 0.1441
	*p*-value	*<0.0001*	*0.0504*	*0.6764*	*0.6434*	*0.9325*	*0.9091*	*0.4571*

Values are presented as regression coefficients (β) with 95% confidence intervals. Unstandardized β coefficients are expressed in the units shown in the table, whereas standardized β coefficients are unitless. Models were adjusted for sex (0 = female, 1 = male), age, energy intake (kcal/day), BMI (kg/m^2^), and IPAC. TIQ, Total Intelligence Quotient; VCI, Verbal Comprehension Index; PRI, Perceptual Reasoning Index; WMI, Working Memory Index; PSI, Processing Speed Index. Italic values indicate the 95% confidence intervals for the corresponding regression coefficients. β_₀_ represents the intercept, and β_₁_–β_₆_ correspond to the regression coefficients for sex, age, energy intake, BMI, IPAC, and selenium intake, respectively.

**Table 7:** Multiple linear regression models for associations between serum selenium and cognitive outcomes.

Outcome	Units	β_**0**_	β_**1**_	β_**2**_	β_**3**_	β_**4**_	β_**5**_	β_**6**_
		Not applicable	Categorical	Per year	Per kcal/day	Per kg/m^2^		Per μg/L
		Intercept	Sex	Age	Energy	BMI	IPAC	Serum selenium
TIQ	Unstandardized estimate	93.82	9.474	−0.7441	0.001982	0.02898	−1.269	0.03062
*R*^2^ = 0.2653	95% CI	73.31 to 114.3	4.478 to 14.47	−1.536 to 0.04746	−0.001607 to 0.005570	−0.4771 to 0.5351	−5.182 to 2.643	−0.07954 to 0.1408
*n* = 132	Standardized estimate	−2.522E-16	0.4381	−0.1561	0.1051	0.009461	−0.04985	0.05591
	95% CI	−0.1512 to 0.1512	0.2071 to 0.6692	−0.3222 to 0.009957	−0.08518 to 0.2953	−0.1557 to 0.1747	−0.2035 to 0.1038	−0.1452 to 0.2571
	*p*-value	*<0.0001*	*0.0003*	*0.0652*	*0.2765*	*0.9099*	*0.522*	*0.5832*
VCI	Unstandardized estimate	95.72	13.79	−0.6636	0.002489	−0.1176	−2.561	0.08514
*R*^2^ = 0.3881	95% CI	72.33 to 119.1	8.094 to 19.49	−1.567 to 0.2392	−0.001604 to 0.006582	−0.6949 to 0.4596	−7.023 to 1.901	−0.04050 to 0.2108
*n* = 132	Standardized estimate	1.785E-16	0.5103	−0.1114	0.1056	−0.03072	−0.08047	0.1244
	95% CI	−0.1379 to 0.1379	0.2995 to 0.7212	−0.2630 to 0.04016	−0.06804 to 0.2792	−0.1815 to 0.1200	−0.2207 to 0.0597	−0.05917 to 0.3080
	*p*-value	*<0.0001*	*<0.0001*	*0.1483*	*0.231*	*0.6875*	*0.2582*	*0.1823*
WMI	Unstandardized estimate	104.4	12.27	−1.362	0.001434	0.1546	−4.755	0.08855
*R*^2^ = 0.2767	95% CI	77.16 to 131.6	5.639 to 18.90	−2.413 to −0.3109	−0.003330 to 0.006197	−0.5173 to 0.8265	−9.949 to 0.4390	−0.05769 to 0.2348
*n* = 132	Standardized estimate	9.174E-18	0.4241	−0.2135	0.05681	0.03772	−0.1396	0.1209
	95% CI	−0.1500 to 0.1500	0.1949 to 0.6534	−0.3783 to −0.04875	−0.1320 to 0.2456	−0.1262 to 0.2016	−0.2920 to 0.0128	−0.07873 to 0.3204
	*p*-value	*<0.0001*	*0.0004*	*0.0115*	*0.5525*	*0.6496*	*0.0724*	*0.233*
PRI	Unstandardized estimate	94.05	6.444	−0.5618	0.001512	0.07445	2.554	−0.01609
*R*^2^ = 0.09305	95% CI	67.84 to 120.3	0.05873 to 12.83	−1.573 to 0.4499	−0.003074 to 0.006098	−0.5724 to 0.7213	−2.446 to 7.555	−0.1569 to 0.1247
*n* = 132	Standardized estimate	−2.579E-16	0.2591	−0.1025	0.06969	0.02113	0.0872	−0.02554
	95% CI	−0.1679 to 0.1679	0.002361 to 0.5157	−0.2870 to 0.08205	−0.1417 to 0.2811	−0.1624 to 0.2047	−0.0834 to 0.2579	−0.2490 to 0.1980
	*p*-value	*<0.0001*	*0.048*	*0.2739*	*0.5153*	*0.8202*	*0.3139*	*0.8215*
PSI	Unstandardized estimate	86.57	−4.223	0.3434	0.000371	0.006993	−0.08873	−0.07986
*R*^2^ = 0.05636	95% CI	59.26 to 113.9	−10.88 to 2.432	−0.7110 to 1.398	−0.004409 to 0.005151	−0.6672 to 0.6811	−5.300 to 5.123	−0.2266 to 0.06687
*n* = 132	Standardized estimate	1.642E-16	−0.1661	0.0613	0.01673	0.001942	−0.002964	−0.1241
	95% CI	−0.1713 to 0.1713	−0.4280 to 0.09569	−0.1269 to 0.2495	−0.1989 to 0.2323	−0.1853 to 0.1892	−0.1771 to 0.1711	−0.3520 to 0.1039
	*p*-value	*<0.0001*	*0.2115*	*0.5203*	*0.8782*	*0.9837*	*0.9732*	*0.2835*

Values are presented as regression coefficients (β) with 95% confidence intervals. Unstandardized β coefficients are expressed in the units shown in the table, whereas standardized β coefficients are unitless. Models were adjusted for sex (0 = female, 1 = male), age, energy intake (kcal/day), BMI (kg/m^2^), and IPAC. TIQ, Total Intelligence Quotient; VCI, Verbal Comprehension Index; PRI, Perceptual Reasoning Index; WMI, Working Memory Index; PSI, Processing Speed Index. Italic values indicate the 95% confidence intervals for the corresponding regression coefficients. β_₀_ = intercept; β_₁_ = sex (0 = female, 1 = male); β_₂_ = age (years); β_₃_ = energy intake (kcal/day); β_₄_ = BMI (kg/m^²^); β_₅_ = IPAC score; β_6_ = serum selenium concentration (µg/L).

Table 7 shows the multiple linear regression models evaluating the association between serum selenium and cognitive outcomes. After adjustment for sex, age, energy intake, BMI, and IPAC, serum selenium was not significantly associated with TIQ, VCI, WMI, PRI, or PSI. Although the direction of the coefficients was positive for TIQ, VCI, WMI, and PRI in the unstandardized models, none of these associations reached statistical significance. For PSI, serum selenium also showed no significant association. Among the covariates, sex remained significantly associated with TIQ and VCI, and energy intake was positively associated with TIQ. Overall, these findings suggest that serum selenium was not an independent predictor of cognitive performance in the adjusted models.

Model comparison based on AICc (Akaike Information Criterion corrected) indicated that the preferred selenium metric varied across cognitive domains. For PSI, the serum selenium model was preferred over the dietary intake model, with a lower AICc (ΔAICc = 0.63). For VCI, the serum selenium model was also preferred (ΔAICc = 1.83). In contrast, dietary selenium intake was favored for TIQ (ΔAICc = 4.41), PRI (ΔAICc = 10.81), and WMI (ΔAICc = 0.18). In summary, dietary selenium intake was favored for TIQ, PRI, and WMI, whereas serum selenium was favored for VCI and PSI. In the adjusted regression models, dietary selenium intake was significantly inversely associated with TIQ and PRI, while serum selenium was not significantly associated with any cognitive outcome. Overall, these findings suggest that dietary selenium intake better captured variation in several cognitive domains, whereas serum selenium provided a better fit for VCI and PSI.

## 4 Discussion

This study examines the relationship between selenium dietary intake and serum selenium levels in young university students and their association with intellectual and cognitive performance, given that this antioxidant micronutrient plays a key role in synaptic plasticity as well as in intellectual and cognitive performance.

Overall, our findings indicate that selenium nutritional status is related to cognitive outcomes in this cohort. Students with low-average intellectual performance (TIQ < 90) exhibited lower serum selenium concentrations than those with TIQ ≥ 90. Beyond global intellectual performance, lower selenium intake and/or serum selenium concentrations were also observed among students with lower performance in specific cognitive domains, particularly verbal comprehension (VCI) and working memory (WMI). In addition, dietary selenium intake was positively correlated with serum selenium concentration, and both intake and serum selenium were positively associated with TIQ and VCI, while serum selenium was also associated with WMI. Collectively, these results support an association between selenium exposure (intake and biomarker status) and intellectual as well as domain-specific cognitive performance in university students.

Notably, however, multivariable analyses suggested a more complex pattern. After adjustment for sex, age, IPAC, BMI and energy intake, dietary selenium intake was not positively associated with VCI, WMI, or PSI and showed an inverse association with TIQ and PRI. This discrepancy between unadjusted and adjusted results may reflect residual confounding and the strong correlation between selenium intake and overall energy intake and dietary patterns, as well as measurement error related to dietary under-reporting. Moreover, measurement error in self-reported dietary intake may further attenuate associations and obscure non-linear patterns (48). It is also possible that selenium-cognition associations are non-linear and that group-level comparisons and correlations may mask heterogeneous effects across subgroups.

Visual smoothers (LOWESS/splines) suggested mild curvature in the overall sample, but this was not consistent after sex stratification. Moreover, we examined residual plots from the linear, LOWESS, and spline fits. These plots did not reveal a clear systematic pattern, suggesting that any non-linear component of the association between serum selenium, or selenium intake, and cognitive indices is likely modest.

Non-linear patterns were not clearly observed in exploratory analyses. However, this absence should be interpreted cautiously, as the limited variability in selenium status within this young cohort may reduce the sensitivity to detect non-linear associations (49).

Therefore, the present findings should be interpreted as associations rather than evidence of causality, and future longitudinal studies and controlled interventions are needed to clarify the independent role of selenium intake and status in cognitive performance among university students.

Regarding participant characteristics, the study population comprises generally healthy student, although with a sedentary lifestyle and little sports practice, mainly among women. This trend coincides with recent studies in the Spanish university population, which describe low levels of physical activity, high sedentary time and a worse profile of sports practice in women compared to men (50–52). Furthermore, comparative European studies have shown that Spanish university students exhibit particularly high levels of sedentary behavior compared to their counterparts in other countries (53).

In our sample, excess body weight was notably frequent among male students (45.9%), in line with findings in young adults from Madrid (54) and higher than estimates reported in first-year Spanish university cohorts (UniHcos), where the prevalence among male has been described around 25–28% (55). This prevalence also appears higher than that described in some cohorts of Australian biomedical students, where excess body weight has been reported around 20–30% (56). Although these characteristics do not establish causation, they provide important context because lifestyle factors such as physical activity, dietary patterns, and body composition may confound or modify associations between micronutrient status and cognition.

On average, reported energy intake was lower than estimated energy expenditure, with a mean relative difference of 1.6% suggesting possible under-reporting of dietary intake, a well-known limitation of self-reported dietary assessment, especially in young and adult populations. Our observations are consistent with earlier work indicating that university students may modify responses to dietary assessments due to social, cultural, or psychological influences and perceived social desirability (57–59). This limitation should be considered when interpreting associations involving dietary selenium intake, particularly in multivariable models that also include energy intake. Nevertheless, the positive correlation between selenium intake and serum selenium supports internal consistency between reported intake and biomarker status.

In terms of selenium exposure, mean dietary selenium intake in our sample was adequate and higher than values reported in healthy Spanish adults (75.0 ± 1 μg/day) (60) and in Spanish children (91 ± 1 μg/day) (18), while it was lower than values reported in Spanish adolescents from the ENALIA study (131.3 ± 24 μg/day) (24). Intake levels in our cohort were also higher than those described in healthy Iranian individuals older than 16 years (67 μg/day in male and 62.1 μg/day in female) (61). Our findings are also consistent with previous research suggesting that cognitive performance in university students may relate to healthier dietary patterns and adequate micronutrient intake (62–64). Importantly, sex differences in selenium intake observed in this study likely reflect differences in consumption of major selenium-contributing food groups, particularly meat-fish-eggs and cereals/pulses, which are typically key dietary sources of selenium, although selenium content may vary with animal feeding practices and soil selenium availability for crops (65, 66).

Mean serum selenium concentrations in this university cohort were within reference values and comparable to those reported in other young populations, including Australian university female (87.6 μg/L) (67) and some adolescent cohorts (68, 69) and they were similar to values reported in Iranian populations (100.6 ± 13 μg/L) (61). Compared with Spanish adult cohorts, our serum levels were in the range of values previously reported (70–72) and higher than those described in some child cohorts (18). We also observed higher serum selenium in male than in female, consistent with previous findings (61). This difference may reflect higher selenium intake among male in our cohort, and may also relate to sex-specific biological factors, as selenium is involved in sperm mitochondrial capsule proteins and may have structural roles in male reproductive physiology (73). Of note, although mean serum selenium values were within reference ranges, a substantial proportion of female (53.5%) and 16.4% of male fell below the reference value (<80 μg/L). This categorization reflects serum selenium concentrations relative to a functional adequacy threshold and should not be interpreted as a clinical diagnosis of selenium deficiency, as Thomson (43) notes that the concentration at which deficiency is likely to occur below 1.0 μmol/L remains unresolved due to the lack of robust clinical markers. This apparent contrast can occur when variability is high and when status is summarized using a single threshold; it highlights that group means may mask potentially relevant subgroups with lower selenium status. Lower serum selenium concentrations may reflect differences in intake and/or physiological factors affecting selenium metabolism and distribution (74).

The observed associations between selenium status and cognition are consistent with a growing body of evidence linking adequate selenium status to neurodevelopment and cognitive function, potentially through antioxidant and neuroprotective pathways (23, 75). In adolescents, higher serum selenium concentrations have been associated with better scores in cognitive function tests, working memory, attention, and academic-related outcomes (68, 69, 76). Despite the specific relevance of selenium for cognition in young people, evidence in university populations remains limited; to our knowledge, research in higher education settings is scarce, with only one study reported in this context (67). More broadly, prior work suggests that blood micromineral levels, including selenium, may be relevant to brain maturation and neurocognitive functioning during youth, and that multiple micronutrient deficiencies may relate to attention, concentration, visuomotor coordination, and working memory (77–79). Mechanistically, selenium acts through selenoproteins, which have been implicated in neuronal function and redox homeostasis, including roles in GABAergic interneurons and neurobiological processes relevant to learning and memory (9, 80, 81). In addition, selenium status has been linked to selenoprotein P, hippocampal neurogenesis, and cortical features in executive-related regions across different stages of life (9, 82, 83). While these pathways provide biological plausibility, the present results, particularly the adjusted models, underscore the need for cautious interpretation and for further research to disentangle selenium-specific effects from broader dietary and lifestyle correlates.

Several limitations should be acknowledged. First, the sample size was modest and derived from a single Spanish university, limiting generalizability to the broader university population. Second, the cross-sectional design precludes causal inference and directionality. Third, dietary intake was assessed via self-report and may be affected by under-reporting, which could attenuate true associations and influence multivariable estimates. Fourth, although we examined multiple cognitive outcomes and indices, findings should be interpreted cautiously in light of multiple comparisons and potential residual confounding (e.g., overall diet quality, sleep, stress, socioeconomic factors, physical activity, and body composition). Future longitudinal studies and well-designed nutritional interventions are warranted to clarify the temporal and potentially causal relationships between selenium intake/status and cognitive performance in university students. An additional limitation is the absence of socioeconomic, family, and early educational variables, which are known determinants of cognitive performance (84, 85). These factors were not collected in the present study and therefore could not be included as covariates in the regression models. Although the relative homogeneity of the university student population may reduce variability in these characteristics, residual confounding cannot be excluded.

Despite these limitations, this study has notable strengths. Dietary selenium intake was estimated using a four-day dietary record including a holiday, and cognitive function was assessed using the WAIS-IV, a widely accepted gold standard for cognitive assessment in individuals aged 16–90 years. Furthermore, to our knowledge, this is among the first studies in Spanish university students to jointly evaluate dietary selenium intake, serum selenium concentrations, and their associations with TIQ and specific cognitive domains.

## 5 Conclusion

In conclusion, in this cohort of young university students, selenium intake and serum selenium concentrations were associated with global and domain-specific cognitive performance in unadjusted analyses, whereas multivariable models indicated a more complex pattern. Selenium nutritional status appears improvable in this university population, particularly among female, in whom a substantial proportion of serum selenium values fell below the reference value. Identifying young adults with inadequate intake and/or low serum selenium should be a priority for health education and prevention strategies. However, given the cross-sectional design, causality cannot be inferred; prospective studies and intervention trials are needed to confirm these findings and warranting confirmation of these associations in larger longitudinal studies.

## Data Availability

The raw data supporting the conclusions of this article will be made available by the authors, without undue reservation.

## References

[1] Zhang F Li X Wei Y. Selenium and Selenoproteins in health. Biomolecules. (2023) 13:799–825. doi: 10.3390/biom13050799, 37238669 PMC10216560

[2] Hariharan S Dharmaraj S. Selenium and selenoproteins: it's role in regulation of inflammation. Inflammopharmacology. (2020) 28:667–95. doi: 10.1007/s10787-020-00690-x, 32144521 PMC7222958

[3] Daneshpour A Leite MEN Wagner KH Sabico S Al-Daghri NM Aldisi D . Selenium and brain aging: A comprehensive review with a focus on hippocampal neurogenesis. Ageing Res Rev. (2025) 112:102898. doi: 10.1016/j.arr.2025.102898, 40946974

[4] Minich WB. Selenium metabolism and biosynthesis of Selenoproteins in the human body. Biochemistry. (2022) 87:S168–77. doi: 10.1134/S0006297922140139, 35501994 PMC8802287

[5] Weaver K Skouta R. The Selenoprotein glutathione peroxidase 4: from molecular mechanisms to novel therapeutic opportunities. Biomedicine. (2022) 10:891. doi: 10.3390/biomedicines10040891, 35453641 PMC9027222

[6] Lu J Holmgren A. The thioredoxin antioxidant system. Free Radic Biol Med. (2014) 66:75–87. doi: 10.1016/j.freeradbiomed.2013.07.036, 23899494

[7] Jelinek M Jurajda M Duris K. Oxidative stress in the brain: basic concepts and treatment strategies in stroke. Antioxidants. (2021) 10:1886. doi: 10.3390/antiox10121886, 34942989 PMC8698986

[8] Cobley JN Fiorello ML Bailey DM. 13 reasons why the brain is susceptible to oxidative stress. Redox Biol. (2018) 15:490–503. doi: 10.1016/j.redox.2018.01.008, 29413961 PMC5881419

[9] Bai YZ Zhang Y Zhang SQ. New horizons for the role of selenium on cognitive function: advances and challenges. Metab Brain Dis. (2024) 39:1255–68. doi: 10.1007/s11011-024-01375-y, 38963634

[10] Nicholson JL Toh P Alfulaij N Berry MJ Torres DJ. New insights on selenoproteins and neuronal function. Free Radic Biol Med. (2022) 190:55–61. doi: 10.1016/j.freeradbiomed.2022.07.021, 35948259

[11] Nazıroğlu M Öz A Yıldızhan K. Selenium and neurological diseases: focus on peripheral pain and TRP channels. Curr Neuropharmacol. (2020) 18:501–17. doi: 10.2174/1570159X18666200106152631, 31903884 PMC7457405

[12] Naderi M Puar P Zonouzi-Marand M Chivers DP Niyogi S Kwong RWM. A comprehensive review on the neuropathophysiology of selenium. Sci Total Environ. (2021) 767:144329. doi: 10.1016/j.scitotenv.2020.144329, 33445002

[13] Rayman MP. Selenium and human health. Lancet. (2012) 379:1256–68. doi: 10.1016/S0140-6736(11)61452-9, 22381456

[14] Barchielli G Capperucci A Tanini D. The role of selenium in pathologies: an updated review. Antioxidants. (2022) 11:251. doi: 10.3390/antiox11020251, 35204134 PMC8868242

[15] Mutonhodza B Chagumaira C Dembedza MP Joy EJM Manzeke-Kangara MG Njovo H . A pilot survey of selenium status and its geospatial variation among children and women in three rural districts of Zimbabwe. Front Nutr. (2023) 10:1235113. doi: 10.3389/fnut.2023.1235113, 37497053 PMC10367098

[16] Gao S Jin Y Hall KS Liang C Unverzagt FW Ji R . Selenium level and cognitive function in rural elderly Chinese. Am J Epidemiol. (2007) 165:955–65. doi: 10.1093/aje/kwk073, 17272290 PMC2760949

[17] Galić L Galić V Ivezić V Zebec V Jović J Đikić M . Modelling leverage of different soil properties on selenium water-solubility in soils of Southeast Europe. Agronomy. (2023) 13:824. doi: 10.3390/agronomy13030824

[18] Navia B Ortega RM Perea JM Aparicio A López-Sobaler AM Rodríguez-Rodríguez E. Selenium status in a group of schoolchildren from the region of Madrid. Spain J Hum Nutr Diet. (2014) 27:239–46. doi: 10.1111/jhn.12126, 23679102

[19] Ortega RM Rodríguez-Rodríguez E Aparicio A Jiménez-Ortega AI Palmeros C Perea JM . Young children with excess of weight show an impaired selenium status. Int J Vitamin Nutr Res Internationale Zeitschrift Fur Vitamin. (2012) 82:121–9. doi: 10.1024/0300-9831/a000101, 23065837

[20] Skröder H Kippler M Tofail F Vahter M. Early-life selenium status and cognitive function at 5 and 10 years of age in Bangladeshi children. Environ Health Perspect. (2017) 125:117003. doi: 10.1289/EHP1691, 29116931 PMC5947942

[21] Amorós R Murcia M Ballester F Broberg K Iñiguez C Rebagliato M . Selenium status during pregnancy: influential factors and effects on neuropsychological development among Spanish infants. Sci Total Environ. (2018) 610-611:741–9. doi: 10.1016/j.scitotenv.2017.08.042, 28822941

[22] Amorós R Murcia M González L Rebagliato M Iñiguez C Lopez-Espinosa MJ . Maternal selenium status and neuropsychological development in Spanish preschool children. Environ Res. (2018) 166:215–22. doi: 10.1016/j.envres.2018.06.002, 29890426

[23] Berr C Arnaud J Akbaraly TN. Selenium and cognitive impairment: a brief-review based on results from the EVA study. Biofactors. (2012) 38:139–44. doi: 10.1002/biof.1003, 22419540

[24] López-Sobaler AM Aparicio A González-Rodríguez LG Cuadrado-Soto E Rubio J Marcos V . Adequacy of usual vitamin and mineral intake in Spanish children and adolescents: ENALIA study. Nutrients. (2017) 9:128. doi: 10.3390/nu9020131, 28208814 PMC5331562

[25] European Union. Regulation (EU) 2016/679 of the European Parliament and of the council of 27 April 2016 on the protection of natural persons with regard to the processing of personal data and on the free movement of such data (general data protection regulation). Off J Eur Union Luxembourg. (2016) L119:1–88.

[26] Legislación consolidada. Ley Orgánica 3/2018, de 5 de Diciembre, de Protección de Datos Personales y Garantía de Los Derechos Digitales. (2018). p. 18.

[27] Chobanian AV Bakris GL Black HR Cushman WC Green LA Izzo JL Jr . Seventh report of the joint National Committee on prevention, detection, evaluation, and treatment of high blood pressure. Hypertension. (2003) 42:1206–52. doi: 10.1161/01.HYP.0000107251.49515.c2, 14656957

[28] Piepoli MF Hoes AW Agewall S Albus C Brotons C Catapano AL . 2016 European guidelines on cardiovascular disease prevention in clinical practice: the sixth joint task force of the European Society of Cardiology and Other Societies on cardiovascular disease prevention in clinical practice (constituted by representatives of 10 societies and by invited experts)developed with the special contribution of the European Association for Cardiovascular Prevention & rehabilitation (EACPR). Eur Heart J. (2016) 37:2315–81. doi: 10.1093/eurheartj/ehw106, 27222591 PMC4986030

[29] Mason JW Ramseth DJ Chanter DO Moon TE Goodman DB Mendzelevski B. Electrocardiographic reference ranges derived from 79,743 ambulatory subjects. J Electrocardiol. (2007) 40:228–234.e8. doi: 10.1016/j.jelectrocard.2006.09.003, 17276451

[30] Dempsey JA Wagner PD. Exercise-induced arterial hypoxemia. J Appl Physiol (1985). 1999;87:1997–2006, doi: 10.1152/jappl.1999.87.6.1997, . 10601141

[31] Ortega RM Requejo AM López-Sobaler AM. "Activity Questionnaire". In: Ortega RM Requejo AM, editors. Nutriguía. Manual de Nutrición Clínica en Atención Primari*a*. Madrid: Editorial Médica Panamericana (2015)

[32] Institute of Medicine (US). Dietary Reference Intakes for Energy, Carbohydrate, fiber, Fat, Fatty Acids, Cholesterol, Protein, and Amino Acids. Washington, DC: National Academies Press (2005).

[33] WHO /FAO/ /UNU Expert Consultation. Energy and protein requirements. World Health Organ Tech Rep Ser. (1985) 724:1–206. 3937340

[34] Stewart A Ackland T. "Anthropometry in physical performance and health". In: Body Composition. Boca Raton, FL: CRC Press (2017). p. 89–108.

[35] Salas-Salvadó J Rubio MA Barbany M Moreno B. SEEDO 2007 consensus for the evaluation of overweight and obesity and the establishment of therapeutic intervention criteria. Med Clin (Barc). (2007) 128:184–96. doi: 10.1016/S0025-7753(07)72531-9, 17298782

[36] Lean ME Han TS Deurenberg P. Predicting body composition by densitometry from simple anthropometric measurements. Am J Clin Nutr. (1996) 63:4–14. doi: 10.1093/ajcn/63.1.4, 8604668

[37] Ortega RM Requejo AM López-Sobaler AM. "Registro de consumo de alimentos y bebidas". In: Ortega RM Requejo AM, editors. Nutriguía. Manual de Nutrición Clínica en Atención Primari*a*. Madrid: Editorial Médica Panamericana (2015)

[38] Ortega RM López-Sobaler AM Andrés P Requejo AM Aparicio A Molinero LM. DIAL Software for Assessing Diets and Food Calculations. Version 3.15. Madrid: Department of Nutrition and Food Science (UCM) & Alceingeniería S.A (2021).

[39] Ortega RM López-Sobaler AM Andrés P Aparicio A. Food Nutritional Composition: A Tool for the Design and Evaluation of Foods and Diets. Madrid: Department of Nutrition and Food Science, Complutense University of Madrid (2021).

[40] Ortega RM Requejo AM Navia B López-Sobaler AM Aparicio A. Recommended Daily Intakes of Energy and Nutrients for the Spanish Population: Department of Nutrition and Food Science. Madrid: Faculty of Pharmacy, Complutense University of Madrid (2019).

[41] Ortega Anta RM Quintas E Sánchez B Andrés P Requejo AM Encinas A. Underestimation of energy intake in a group of young female university students of Madrid. Rev Clin Esp. (1997) 197:545–9. 9312790

[42] Labat L Dehon B Lhermitte M. Rapid and simple determination of selenium in blood serum by inductively coupled plasma-mass spectrometry (ICP-MS). Anal Bioanal Chem. (2003) 376:270–3. doi: 10.1007/s00216-003-1881-6, 12677338

[43] Thomson CD. Assessment of requirements for selenium and adequacy of selenium status: a review. Eur J Clin Nutr. (2004) 58:391–402. doi: 10.1038/sj.ejcn.1601800, 14985676

[44] Wechsler D. Escala de Inteligencia de Wechsler Para Adultos-IV (WAIS-IV). Pearson Educación: Madrid (2012).

[45] Ferreira García E Calderón GC. Evaluación de Adultos: Prácticas WAIS-IV. Evaluación de Aptitudes Cognitivas. Madrid ed.Pearson Education (2022).

[46] World Health Organization. WHO Guidelines on Physical Activity and Sedentary Behaviour. Geneva: World Health Organization (2020). 33369898

[47] Calleja CA Hurtado MMC Daschner Á Escámez PSF Abuín CMF Pons RMG . Informe del Comité Científico de la Agencia Española de Seguridad Alimentaria y Nutrición (AESAN) sobre Ingestas Nutricionales de Referencia para la población española. Dialnet. (2019) 29:43–68.

[48] Rutter CE Millard LAC Borges MC Lawlor DA. Exploring regression dilution bias using repeat measurements of 2858 variables in ≤49 000 UK biobank participants. Int J Epidemiol. (2023) 52:1545–56. doi: 10.1093/ije/dyad082, 37336529 PMC10555784

[49] May S Bigelow C. Modeling nonlinear dose-response relationships in epidemiologic studies: statistical approaches and practical challenges. Dose Response. (2006) 3:474–90. doi: 10.2203/dose-response.003.04.004, 18648629 PMC2477199

[50] Acebes-Sánchez J Diez-Vega I Rodriguez-Romo G. Physical activity among Spanish undergraduate students: A descriptive correlational study. Int J Environ Res Public Health. (2019) 16:2770. doi: 10.3390/ijerph16152770, 31382503 PMC6696045

[51] Brandariz JRR-V Varela-Lema L Santiago-Pérez MI Candal-Pedreira C Guerra-Tort C Ruano-Raviña A . Sedentary behavior and physical inactivity from a comprehensive perspective. Gaceta Sanitaria. (2023) 37:54. doi: 10.1016/j.gaceta.2023.102352 38056139

[52] Monserrat-Hernández M Checa-Olmos JC Arjona-Garrido Á López-Liria R Rocamora-Pérez P. Academic stress in university students: the role of physical exercise and nutrition. Healthcare. (2023) 11:2401. doi: 10.3390/healthcare11172401, 37685435 PMC10486982

[53] Paulo R Ramalho A Scursatone I Caire M Calle NB Bores-García D . Comparative study of physical activity, leisure preferences, and sedentary behavior among Portuguese, Italian, and Spanish university students. Healthcare. (2024) 12:1930. doi: 10.3390/healthcare12191930, 39408111 PMC11476021

[54] Iglesias M Mata G Pérez A Hernández S García-Chico R Papadaki C. Estudio nutricional en un grupo de estudiantes universitarios madrileños. Nutr Clín Diet Hosp. (2013) 33:23–30.

[55] Hernández-Segura N Botella-Juan L Amezcua-Prieto C Morales-Suárez-Varela M Mateos-Campos R Fernández-Villa T . Excess weight in relation to lifestyle habits in Spanish first-year university students: differences between pre- and post-COVID-19-A serial cross-sectional study based on uniHcos project. Healthcare. (2023) 11:1547. doi: 10.3390/healthcare11111547, 37297687 PMC10252620

[56] Gallo LA Gallo TF Young SL Fotheringham AK Barclay JL Walker JL . Adherence to dietary and physical activity guidelines in Australian undergraduate biomedical students and associations with body Composition and metabolic health: A cross-sectional study. Nutrients. (2021) 13:3354. doi: 10.3390/nu13103500, 34684500 PMC8538134

[57] Wehling H Lusher J. People with a body mass index ⩾30 under-report their dietary intake: a systematic review. J Health Psychol. (2019) 24:2042–59. doi: 10.1177/1359105317714318, 28810493

[58] Kagawa M Hills AP. Preoccupation with body weight and under-reporting of energy intake in female Japanese nutrition students. Nutrients. (2020) 12:879. doi: 10.3390/nu12030830, 32244995 PMC7146226

[59] Maneschy I Jimeno-Martínez A Miguel-Berges ML Rupérez AI Ortega-Ramiréz AD Masip G . Eating behaviours and dietary intake in children and adolescents: A systematic review. Curr Nutr Rep. (2024) 13:363–76. doi: 10.1007/s13668-024-00544-w, 38797817 PMC11327180

[60] Olza J Aranceta-Bartrina J González-Gross M Ortega RM Serra-Majem L Varela-Moreiras G . Reported dietary intake and food sources of zinc, selenium, and vitamins A, E and C in the Spanish population: findings from the ANIBES study. Nutrients. (2017) 9:697. doi: 10.3390/nu9070697, 28684689 PMC5537812

[61] Safaralizadeh R Kardar GA Pourpak Z Moin M Zare A Teimourian S. Serum concentration of selenium in healthy individuals living in Tehran. Nutr J. (2005) 4:32. doi: 10.1186/1475-2891-4-32, 16285883 PMC1308858

[62] Burrows TL Whatnall MC Patterson AJ Hutchesson MJ. Associations between dietary intake and academic achievement in college students: A systematic review. Healthcare. (2017) 5:63. doi: 10.3390/healthcare5040060 28946663 PMC5746694

[63] Pilato IB Beezhold B Radnitz C. Diet and lifestyle factors associated with cognitive performance in college students. J Am Coll Heal. (2022) 70:2230–6. doi: 10.1080/07448481.2020.1847118, 33320776

[64] Dimas-Benedicto C Albasanz JL Bermejo LM Castro-Vázquez L Sánchez-Melgar A Martín M . Impact of iron intake and reserves on cognitive function in Young university students. Nutrients. (2024) 16:2808. doi: 10.3390/nu16162808, 39203944 PMC11356983

[65] López-Bellido Garrido FJ López BL. Selenium and health; reference values and current status of Spanish population. Nutr Hosp. (2013) 28:1396–406. doi: 10.3305/nh.2013.28.5.6634, 24160192

[66] Authority EFS. Outcome of a Public Consultation on the Draft Guidance of the EFSA Panel on Dietetic Products, Nutrition and Allergies (NDA) on the Scientific Requirements for Health Claims Related to the Immune System, the Gastrointestinal Tract and Defence Against Pathogenic Microorganisms. Wiley Online Library. Report No.: 2397–8325. EFSA: Parma (2016).

[67] Fayet-Moore F Petocz P Samman S. Micronutrient status in female university students: iron, zinc, copper, selenium, vitamin B12 and folate. Nutrients. (2014) 6:5103–16. doi: 10.3390/nu6115103, 25401503 PMC4245582

[68] Rahi B Rashid F Sultana R Benoit J Parvez F Khan K. Impact of nutritional minerals biomarkers on cognitive performance among Bangladeshi rural adolescents-A pilot study. Nutrients. (2024) 16:3865. doi: 10.3390/nu16223865, 39599651 PMC11597576

[69] Saxena R Gamble M Wasserman GA Liu X Parvez F Navas-Acien A . Mixed metals exposure and cognitive function in Bangladeshi adolescents. Ecotoxicol Environ Saf. (2022) 232:113229. doi: 10.1016/j.ecoenv.2022.113229, 35131582 PMC10045507

[70] González-Estecha M Palazón-Bru I Bodas-Pinedo A Trasobares E Palazón-Bru A Fuentes M . Relationship between serum selenium, sociodemographic variables, other trace elements and lipid profile in an adult Spanish population. J Trace Elem Med Biol. (2017) 43:93–105. doi: 10.1016/j.jtemb.2016.12.002, 28073603

[71] Diaz Romero C López Blanco F Henríquez Sánchez P Rodríguez E Serra ML. Serum selenium concentration in a representative sample of the Canarian population. Sci Total Environ. (2001) 269:65–73. doi: 10.1016/S0048-9697(00)00815-9, 11305344

[72] Millán Adame E Florea D Sáez Pérez L Molina López J López-González B Pérez de la Cruz A . Deficient selenium status of a healthy adult Spanish population. Nutricion Hospitalaria. (2012) 27:524–8. doi: 10.1590/S0212-16112012000200026, 22732978

[73] Hansen JC Deguchi Y. Selenium and fertility in animals and man–a review. Acta Vet Scand. (1996) 37:19–30. doi: 10.1186/BF03548116, 8659343 PMC8064001

[74] Prabhu KS Lei XG. Selenium. Adv Nutr. (2016) 7:415–7. doi: 10.3945/an.115.010785, 26980826 PMC4785479

[75] Ferdous KA Knol LL Park HA. Association between selenium intake and cognitive function among older adults in the US: National Health and nutrition examination surveys 2011-2014. J Nutr Sci. (2023) 12:e57. doi: 10.1017/jns.2023.43, 37180486 PMC10173086

[76] Rahman A Rao M Aldughpassi A Jallad R Shaban L. Blood levels of copper, manganese, selenium, and zinc are positively associated with cognitive function and academic performance in adolescents. Front Nutr. (2025) 12:1638283. doi: 10.3389/fnut.2025.1638283, 40777180 PMC12330213

[77] Ackerman CM Courtney SM. Spatial relations and spatial locations are dissociated within prefrontal and parietal cortex. J Neurophysiol. (2012) 108:2419–29. doi: 10.1152/jn.01024.2011, 22896722 PMC3545176

[78] Chai WJ Abd Hamid AI Abdullah JM. Working memory from the psychological and neurosciences perspectives: A review. Front Psychol. (2018) 9:401. doi: 10.3389/fpsyg.2018.00401, 29636715 PMC5881171

[79] Singh S Awasthi S Kumar D Sarraf SR Pandey AK Agarwal GG . Micronutrients and cognitive functions among urban school-going children and adolescents: A cross-sectional multicentric study from India. PLoS One. (2023) 18:e0281247. doi: 10.1371/journal.pone.0281247, 36730336 PMC9894395

[80] Salah ON Abdelraouf ER Abdelhameed MH Dawood AA Hashish AF Kilany A . Assessment of the level of GABA and some trace elements in blood in children who suffer from familial febrile convulsions (2014) 2:68–73. doi: 10.3889/oamjms.2014.012,

[81] Wolosker H Balu DT. D-serine as the gatekeeper of NMDA receptor activity: implications for the pharmacologic management of anxiety disorders. Transl Psychiatry. (2020) 10:184. doi: 10.1038/s41398-020-00870-x, 32518273 PMC7283225

[82] Zhang X Zhong Y He K. The causal effects between selenium levels and the brain cortical structure: A two-sample Mendelian randomization study. Brain Behav. (2023) 13:e3296. doi: 10.1002/brb3.3296, 37904336 PMC10726828

[83] Pitts MW Hoffmann PR Schomburg L. Editorial: selenium and Selenoproteins in brain development, function, and disease. Front Neurosci. (2021) 15:821140. doi: 10.3389/fnins.2021.821140, 35095409 PMC8792733

[84] Bradley RH Corwyn RF. Socioeconomic status and child development. Annu Rev Psychol. (2002) 53:371–99. doi: 10.1146/annurev.psych.53.100901.135233, 11752490

[85] Nisbett RE Aronson J Blair C Dickens W Flynn J Halpern DF . Intelligence: new findings and theoretical developments. Am Psychol. (2012) 67:130–59. doi: 10.1037/a0026699, 22233090

[86] Williams B Mancia G Spiering W Agabiti Rosei E Azizi M Burnier M . 2018 ESC/ESH guidelines for the management of arterial hypertension. Eur Heart J. (2018) 39:3021–104. doi: 10.1093/eurheartj/ehy339, 30165516

[87] Sammito S Böckelmann I. Reference values for time- and frequency-domain heart rate variability measures. Heart Rhythm. (2016) 13:1309–16. doi: 10.1016/j.hrthm.2016.02.006, 26883166

